# Synthesis of Oligonucleotides Carrying Thiol Groups Using a Simple Reagent Derived from Threoninol

**DOI:** 10.3390/molecules170910026

**Published:** 2012-08-24

**Authors:** Sonia Pérez-Rentero, Santiago Grijalvo, Rubén Ferreira, Ramon Eritja

**Affiliations:** 1 Institute for Research in Biomedicine (IRB Barcelona), Baldiri Reixac 10, E-08028 Barcelona, Spain; Email: sonia.perez@irbbarcelona.org (S.P.-R.); santiago.grijalvo@irbbarcelona.org (S.G.); ruben.ferreira@irbbarcelona.org (R.F.); 2 Institute for Advanced Chemistry of Catalonia (IQAC), CSIC, CIBER-BBN Networking Centre on Bioengineering, Biomaterials and Nanomedicine, Jordi Girona 18, E-08034 Barcelona, Spain

**Keywords:** DNA, solid-phase synthesis, threoninol, thiols, gold nanoparticles

## Abstract

Oligonucleotides carrying thiol groups are useful intermediates for a remarkable number of applications involving nucleic acids. In this study, DNA oligonucleotides carrying *tert*-butylsulfanyl (*t*-BuS) protected thiol groups have been prepared. A building block derived from threoninol has been developed to introduce a thiol group at any predetemined position of an oligonucleotide. The resulting thiolated oligonucleotides have been used for the preparation of oligonucleotide conjugates and for the functionalization of gold nanoparticles using the reactivity of the thiol groups.

## 1. Introduction

Modified oligonucleotides containing thiol groups are being used profusely in several fields and especially in the DNA nanobiotechnology field. Thus, thiolated oligonucleotides were first synthesized to facilitate the introduction of biotin, fluorescent compounds and other nonradioactive labels [1,2]. Later, thiolated oligonucleotides were used to produce oligonucleotide-peptide conjugates using post-synthetic protocols [3,4] and oligonucleotide-protein conjugates [5,6,7]. The preparation of conformationally restricted DNA structures were achieved by crosslinking thiolated oligonucleotides by disulfide bond formation [8,9]. More recently, thiolated oligonucleotides were used for the immobilization of DNA onto gold surfaces [10,11,12] and the preparation of DNA-functionalized gold nanoparticles [13,14,15,16].

We are interested in the incorporation of thiol groups into two-dimensional DNA lattices [17] by introducing a single thiol group per DNA unit. Thiol groups have a strong affinity for gold surfaces and as well they can be used for the introduction of non-radioactive labels and peptides functionalized with maleimido or iodo- or bromoacetamide groups. Previous work has demonstrated that bidimensional DNA arrays carrying thiolated nucleobases at the apex of the topological markers can be deposited on gold surfaces [17] or reacted with the appropriate maleimido derivatives to generate functionalized DNA bidimensional arrays [18,19]. We become interested in the preparation of similar building blocks using an inexpensive non-nucleoside residue as starting material. Here, we describe a straightforward synthesis of a threoninol derivative carrying the (*t*-butyldisulfanyl)ethyl group which allows the introduction of a protected thiol group at any position of an oligonucleotide. The utility of the new derivative is demonstrated with the preparation of several oligonucleotide conjugates using the specific reactivity of the thiol group.

## 2. Results and Discussion

### 2.1. Synthesis of the Threoninol Derivatives Needed for Oligonucleotide Synthesis

**Scheme 1:**
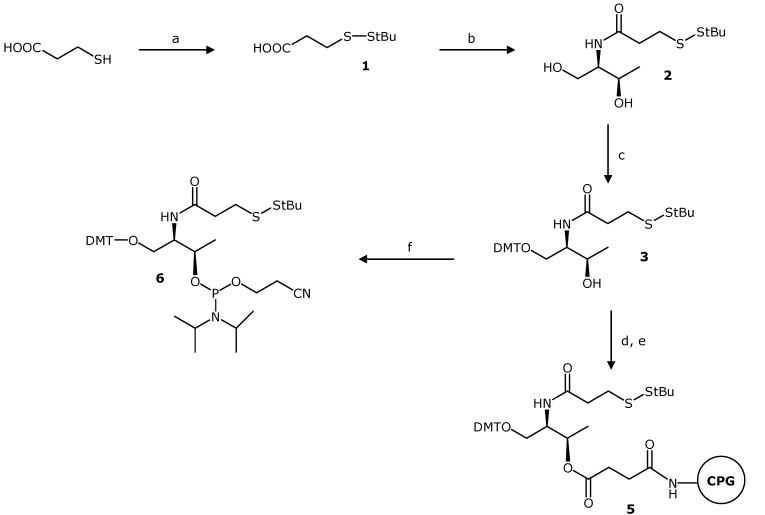
Synthesis of the phosphoramidite and functionalized solid support needed for the synthesis of oligonucleotides bearing thiol groups.

In order to incorporate functional groups at any predetermined position of oligonucleotides it is convenient to select a building block with two hydroxyl groups which would allow the use of the phosphoramidite strategy developed for the synthesis of oligonucleotides. We selected an acyclic scaffold such as L-threoninol to incorporate a 3-mercaptopropionic acid derivative conveniently protected with a *tert*-butylsulfanyl group [4,9,20] into DNA as depicted in Scheme 1. Threoninol has been used previously to introduce several units such as Methyl Red moieties [21], photoactive azobenzenes [22], DNA-binding drugs [23] and tetrathiafulvalene derivatives [24,25] into oligonucleotides. Recently, siRNA derivatives carrying aromatic groups linked through threoninol at their 3'-end were prepared to modulate the binding of a passenger strand to the RISC complex [26]. The *tert*-butylsulfanyl group [4,9,20] was selected for the protection of the thiol group. Other groups such as the *S*-trityl [1], acetyl [27], benzoyl [28], 2,4-dinitrophenylethyl [29], *N*-trifluoroacetamidoethylsulfanyl [30] and 2-nitrobenzyl [31] have been described for the preparation of thiolated oligonucleotides. We selected this protecting group because is compatible with the oligonucleotide synthesis conditions and can be removed in the presence of reducing agents independently of the final ammonia deprotection.

3-Mercaptopropionic acid was reacted with di-*tert*-butyl 1-(*tert*-buylthio)-1,2-hydrazine-dicarboxylate [20] to yield the *t*-BuS-protected derivative **1**. The amino group of threoninol was reacted with the *t*-BuS-protected 3-mercaptopropanoic acid derivative yielding the threoninol amide **2** (Scheme 1). Then, the primary hydroxyl group was protected with the 4,4'-dimethoxytrityl (DMT) group. Finally, the secondary alcohol was used to prepare either the phosphoramidite **6** or the hemisuccinate **4**. The hemisuccinate was further used to functionalize controlled pore glass (CPG) solid support (**5**). Derivatives **5** and **6** were used for the assembly of oligonucleotides using solid-phase methods [25,26].

### 2.2. Oligonucleotide Synthesis

**Figure 1:**
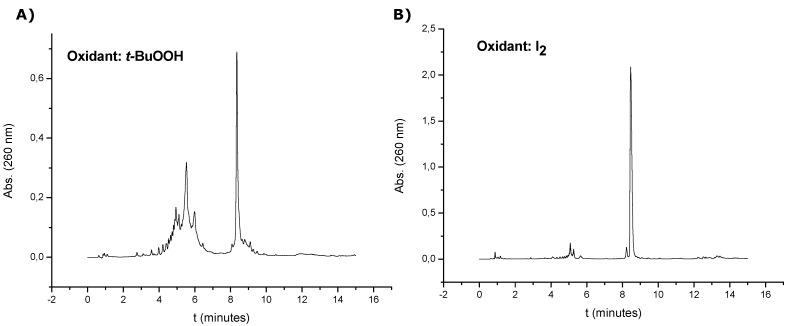
HPLC profiles of crude oligonucleotide **7** using different oxidants: (**A**) 10% *tert*-butyl hydroperoxide solution in ACN and (**B**) standard 0.02 M iodine solution. In both cases ammonia cleavage was performed at r. t. for 1 h.

Oligonucleotide sequences (Table 1) carrying the disulfide group at 3' and 5'-ends as well as in internal positions were assembled using the appropriate chemicals including the solid support **5** and phoshoramidite **6**. Previous work described the oxidation of the *t*-BuS group on a 5-methylcytidine derivative when standard iodine solution was used [19]. This side reaction yielded sulfonic acid derivatives and it was minimized by replacing the commercially available iodine solution used for the phosphite oxidation by a *tert*-butyl hydroperoxide solution [19]. For this reason we first analyzed the effect of both solutions on the stability of the disulfide moiety. Then two syntheses of oligothymidine **7** were carried out. One was achieved using a 10% *tert-*butyl hydroperoxide solution in ACN and the other one using the standard 0.02 M iodine solution. Figure 1 shows the HPLC profiles obtained after ammonia cleavage (1 h, room temperature). In both cases a main peak was observed (t_R_ = 8.0 min), which was collected and characterized by mass spectrometry, corresponding to oligonucleotide **7**. In addition some extra peaks between 4–6 min were observed in the HPLC profiles, especially when the *tert*-butyl hydroperoxide solution was used as oxidant. These products were assigned to degradation products deriving from the oxidation of the *t*-BuS group to sulfonic acids [19] although we could not obtain a clear mass spectrum from this HPLC fraction. We concluded that the best result was obtained when the iodine solution was used as oxidant. For this reason, standard iodine oxidation conditions were used for the preparation of the rest of the DNA sequences (Table 1).

**Table 1:** Oligonucleotide sequences prepared in this study.

#	Sequence (5'-3') ^1^	HPLC (min) ^2^	MS, (expected)	MS, [M−H]^−^ (found)
**7**	TTTTTTTTTTTT- **X(*t-*Bu)**	8.5	3,931.6	3,931.4
**8**	AAAAGGGCAAGG- **X(*t-*Bu)**	8.0	4,095.7	4,093.1
**9**	TTCCA- **X(*t-*Bu)**-ATTACCG	6.5	3,923.6	3,925.4
**10**	**X(*t-*Bu)**-ACTATCCAGGAAA	7.7	4,005.7	4,004.6
**11**	CTGTTGGCTT- **X(*t-*Bu)**-TGCCAACAG	6.1 ^3^	6,134.0	6,132.8
**12**	GCAGTCGCACGACCTGGCGTCTGTTGGCTT- **X(*t-*Bu)**-TGCCAACAGTTTGTACTACGCAATCCTGCCGTATCGACG	6.3 ^3^	21,513.9	A^−12^: 1,792.9 ^4^
**13**	CGGAGGTACATTCGACTTGA- **X(*t-*Bu)**	7.8	6,496.0	6,487.3
**14**	Fluoresceine-CGGAGGTACATTCGACTTGA- **X(*t-*Bu)**	6.1	7,019.0	7,023.8
**15**	AAAAGGGCAAGG- **X**	4.5	4,007.6	3,995.6
**16**	AAAAGGGCAAGG- **X(Nan)**	5.9	4,201.8	4,207.9
**17**	AAAAGGGCAAGG- **X(Py)**	10.8,11.0	4,302.9	4,307.8
**18**	TTTTTTTTTTTT- **X**	5.4	n.d.	n.d.
**19**	TTTTTTTTTTTT- **X(Nan)**	6.6	4,037.7	4,041.4
**20**	CGGAGGTACATTCGACTTGA- **X**	4.7	n.d.	n.d.
**21**	Fluoresceine-CGGAGGTACATTCGACTTGA- **X**	5.6	n.d.	n.d.

^1^ **X(*t-*Bu)** = *N*-[2-(tert-butyldisulfanyl)ethylcarbonyl]-L-threoninol; **X** = *N*-(mercaptoethyl-carbonyl)-L-threoninol; **X(Nan)** = 2-hydroxy-5-nitroacetaniline derivative; **X(Py)** = Pyrenyl maleimido derivative (see Scheme 2). The underlined bases in sequences **11** and **12** are involved in the formation of the hairpin; ^2^ Retention time values obtained in the HPLC analysis (see conditions in experimental part); ^3^ Temperature of the HPLC column was 60 °C to avoid secondary structures; ^4^ mass measured from the M^12−^ by electrospray (Synapt HDMS system); n.d.: not determined.

Oligonucleotides **7** and **8** bearing the disulphide group at the 3'-end position were prepared using the functionalized solid support **5**. The phosphoramidite **6** was used to prepare oligonucleotides **9** and **10** carrying the disulfide moiety in the middle of the sequence and at the 5'-end position respectively. In all cases the modified oligonucleotides **7**–**10** were obtained as the major compounds (Figure S1, supplementary information). The main products were collected and analyzed by mass spectrometry (MALDI-TOF). The results are summarized in Table 1. In addition to the expected compound we have observed the presence of variable amounts of side compounds with shorter retention time (t_R_ around 4 min). When oligonucleotide **7** (prepared with iodine) was treated with the ammonia solution for several hours at 55 °C we observed as well the presence of these side products in the HPLC profiles (data not shown). Then we reasoned that these side compounds are related with the ammonia treatment required for the cleavage and deprotection of nucleobases and phosphate groups. We observed as well that the extension of the side reaction depended on the position where the modified residue was placed. For instance, the amount of these side products detected in the HPLC profile of crude compound **9** (threoninol derivative located in the middle of the sequence), represented less than 5%. On the other hand, HPLC profiles of crude oligonucleotides **8** and **10** (threoninol derivative attached at the ends of each sequence) showed higher amounts (15–25%). MS analysis of the main side compound showed the loss of 104 mass units from the expected molecular mass. We hypothesized that this loss may be due to the removal of the *t*-BuSS group (121 mass units) and to the subsequent addition of a NH_2_ group. This compound may be formed by direct nucleophilic displacement of the *t*-BuSS group or by β-elimination of the *t*-BuSS group followed by ammonia addition to the resulting olefin (Scheme S1, supplementary information). This side reaction was produced when the treatment with ammonia was extended to 12 h at 55 °C for the removal of the isobutyryl group of guanine and it was mainly observed in oligonucleotides carrying the disulfide group at terminal positions. The use of milder protecting groups for guanine such as dimethylformamidino group may help to avoid the elimination of the *t*-BuSS group.

**Scheme 2:**
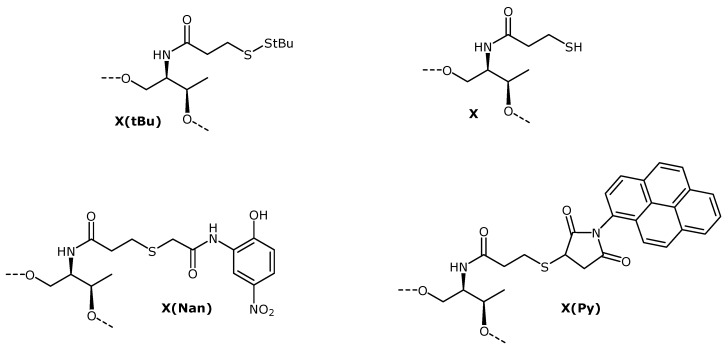
Structures of the threoninol derivatives prepared in this study. The broken lines indicate H or the oligonucleotide chain.

Next we synthesized two long hairpin oligonucleotides **11**,**12** carrying one disulfide group in the middle of the loop (Table 1, Scheme 2). Oligonucleotide **12** corresponds to one of the five oligonucleotides described by the group of Seeman for the preparation of bidimensional DNA arrays by the “tile system” [32]. Specifically this sequence carries a topological marker in the form of a protruding DNA hairpin which has been used to incorporate thiol groups able to anchor bidimensional DNA arrays into gold surfaces [17]. Oligonucleotide **11** corresponds to the protruding hairpin part of oligonucleotides **12** without the single-stranded parts which pair with the corresponding complementary sequences designed to form a DNA tile. Both thiolated oligonucleotides were prepared using the phosphoramidite derivative **6** obtaining the desired compounds. The characterization of oligonucleotide **12** was carried out by electrospray mass spectrometry (Synapt HDMS system, Waters-Micromass). The molecular mass of such a long oligonucleotide was obtained from the A^−12^ (1,792.9) and A^−11^ (1,958.5) ions.

Finally we have prepared oligonucleotides **13** and **14**. These oligonucleotides are 20 mer that will be used for the functionalization of gold nanoparticles. Both oligonucleotides have the same DNA sequence and carry the threoninol derivative at the 3'-end but, in addition, oligonucleotide **14** bears a fluoresceine derivative at the 5'-end to quantify the degree of functionalization of the resulting nanoparticles. Both oligonucleotides were obtained in good yields. Additionally, we cleaved the *t*-BuS group of both disulphide oligonucleotides to obtain the corresponding thiol oligonucleotides **20** and **21**. Preparing both disulfide and thiol analogues allow for comparison of stability and surface coverage of the resulting conjugates. For comparison purposes we have also prepared the 20-mer oligonucleotide sequences carrying the commercially available 3'-thiol modifier (see Supplementary Information).

### 2.3. Denaturation Studies on Duplexes Carrying the Tert-Butylsulfanyl Threoninol Derivative

In order to assess the influence of the threoninol derivative in the duplex structure, we performed melting experiments with the modified oligonucleotides **8**, **9** and **10**. Table 2 shows the melting temperatures of several duplexes carrying the *tert*-butylsulfanyl protected threoninol derivative and the corresponding non modified duplexes. As expected, the presence of the threoninol derivative either at the 3'-end (entry 1) or at the 5'-end (entry 3) of a duplex had very little effect in the stability compared with the corresponding non modified duplex (entries 2 and 4 respectively). The comparison of entries 4 and 5 indicates that the introduction of the *tert*-butylsulfanyl protected threoninol derivative in the middle of the DNA sequence induces a remarkable destabilizing effect (6.3 °C). 

Finally the effect of the modification placed in the middle of the loop of the hairpin **11** was studied. To this end, an unmodified version of the hairpin was synthesized replacing the threoninol derivative by a thymidine. The melting temperatures obtained from the denaturation curves were very similar in both cases (entries 7 and 8). Then we conclude that the presence of the threoninol derivative in the loop do not induce any significant change in the denaturation process of the hairpin.

**Table 2:** Melting temperatures of duplexes carrying the *tert*-butylsulfanyl threoninol derivative.

Entry	Duplex	T_m_ (°C)	ΔT_m_ (°C)
1	5'-AAAAGGGCAAGG-**X(*t*-Bu)**-3' (**8**)/3'-TTTTCCCGTTCC-5'	52.3 ^1^	−0.7
2	Unmodified 5'-AAAAGGGCAAGG-3'/3'-TTTTCCCGTTCC-5'	53.0 ^1^	--
3	5'-**X(*t*-Bu)**-ACTATCAGGAAA-3' (**10**)/3'-TGATAGTCCTTT-5'	43.9 ^1^	+0.5
4	Unmodified 5'-ACTATCAGGAAA-3'/3'-TGATAGTCCTTT-5'	43.4 ^1^	--
5	5'-TTCCA-**X(*t*-Bu)**-ATTACCG-3' (**9**)/3'-AAGGTTAATGGC-5'	42.2 ^1^	−6.3
6	Unmodified 5'-TTCCAATTACCG-3'/3'-AAGGTTAATGGC-5'	48.5 ^1^	--
7	Hairpin 5'-CTGTTGGCTT-**X(*t*-Bu)**-TGCCAACAG-3' (**11**)	75.2 ^2^	−0.2
8	Unmodified hairpin 5'-CTGTTGGCTTTTGCCAACAG-3'	75.4 ^2^	--

^1^ Conditions: 0.3 M NaCl, 10 mM phosphate buffer pH 7.0; ^2^ conditions: 50 mM NaCl, 10 mM phosphate buffer pH 7.0; ΔT_m_ is thedifference in T_m_ between the non modified duplex and the corresponding modified duplex. The underlined bases in sequence **11** are involved in the formation of the hairpin.

### 2.4. Functionalization of Thiol Oligonucleotides

Oligonucleotides bearing thiol groups are important intermediates for the introduction of fluorescent compounds and other non-radioactive labels into oligonucleotides [1,2]. To this end, we studied the preparation of oligonucleotide conjugates by reaction of the thiolated oligonucleotides with maleimido and 2-bromoacetamide compounds. Oligonucleotides **7** and **8** were used to perform reactions with *N*-(1-pyrenyl)maleimide and 2-bromo-2'-hydroxy-5'-nitroacetaniline. The first step consisted in the cleavage of the *t*-BuS group. An aqueous solution of oligonucleotide **8** was treated with a solution of tris(2-carboxyethyl)phosphine (TCEP) at 55 °C. The removal of the *t*-BuS group was monitored by HPLC (Figure S4, supporting information). Under these conditions the complete deprotection of the thiol was achieved in four hours. The resulting oligonucleotide **15** was isolated and characterized by mass spectrometry (Table 1).

**Figure 2:**
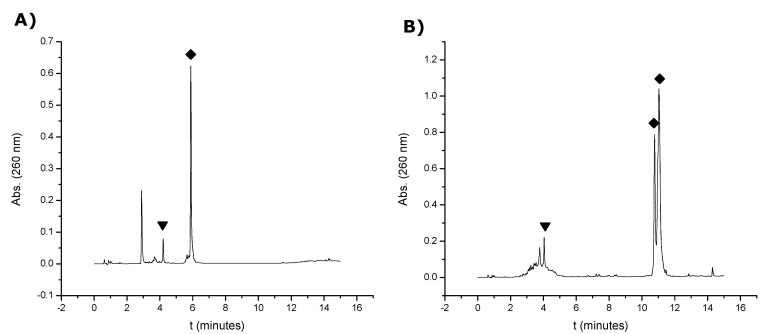
HPLC profiles of the mixtures obtained after the reaction of oligonucleotide **15** with (**A**) 2-bromo-2'-hydroxy-5'-nitroacetaniline to yield compound **16** and (**B**) with *N*-(1-pyrenyl)maleimide to yield compound **17**. (▼ stands for the thiol oligonucleotide **15** and ♦ stands for the resulting oligonucleotide conjugates).

Then, oligonucleotide **15** was reacted either with 2-bromo-2'-hydroxy-5'-nitroacetaniline and *N*-(1-pyrenyl)maleimide overnight. Both conjugates (**16** and **17**) were obtained in good yields, ranging from 80 to 85% as depicted in Figure 2 (conjugation yields were determined by HPLC analysis of the crudes). In both cases the main compounds were collected and analyzed by mass spectrometry. The results are summarized in Table 1. The reaction with *N*-(1-pyrenyl)maleimide yielded two main products (Figure 2B) sharing the same mass once analyzed by mass spectrometry. These compounds may correspond to the two possible isomers coming from the reaction of the thiol group with the maleimide group.

A conjugation assay was performed as well with the oligothymidine thiol sequence. Oligonucleotide **7** once treated with a TCEP solution yielded the deprotected oligonucleotide **18**, which was reacted with 2-bromo-2'-hydroxy-5'-nitroacetaniline. The desired conjugate **19** was obtained in good yield (81%, conjugation yield was determined by HPLC analysis of the crude, Figure S5 supplementary information).

### 2.5. Nanoparticle Functionalization and Stability Measures

**Figure 3:**
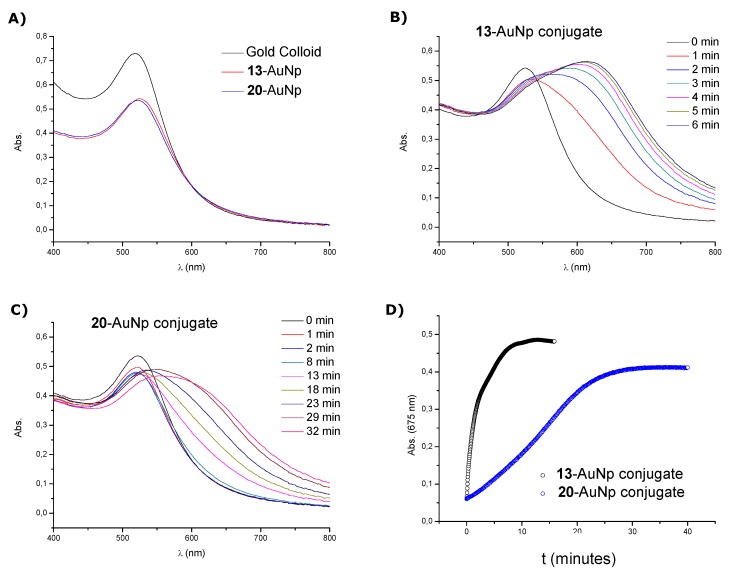
(**A**) UV-vis spectra of the initial gold nanoparticle solution and the functionalized gold nanoparticles with oligonucleotides **13** and **20**. Time evolution of UV-Vis spectra of gold nanoparticle conjugates in 10 mM DTT. (**B**) Disulfide oligonucleotide **13**. (**C**) Thiol oligonucleotide **20**. (**D**) Absorbance changes monitored with time at 675 nm for both conjugates.

We studied as well the functionalization of gold nanoparticles with thiolated oligonucleotides. For this purpose, oligonucleotide **13**, carrying a disulfide modification at the 3'-end was prepared. The thiol oligonucleotide (compound **20**) was prepared by treating oligonucleotide **13** with TCEP as described above. Immobilization of the thiolated oligonucleotides on 10 nm citrate-reduced gold nanoparticles was carried out using standard protocols [33]. The resulting functionalized gold nanoparticles were characterized by UV-vis spectroscopy (Figure 3A). After modification only a small shift in the surface plasmon band was observed (λ_max_ 519 to 525 for **13**-AuNp and λ_max_ 519 to 522 for **20**-AuNp). The effect of the disulfide and thiol modified oligonucleotides on the stability of the conjugates was investigated following procedures described in the literature [34,35]. Each conjugate were treated with DTT (10 mM) at 40 °C and left to aggregate and the UV-vis spectra were recorded at 1 min interval (Figure 3B,C). Stability was evaluated by measuring the increment of absorbance at 675 nm that resulted from gold nanoparticle aggregation (Figure 3D). The half-time t_1/2_ (the time required to reach half the value for complete aggregation) was calculated from the curves. This parameter was determined by taking the midpoint in the absorbance change at 675 nm as a function of time. For conjugates obtained with oligonucleotide **13** (**13**-AuNp) the t_1/2_ was less than 2 min and for the conjugates obtained with oligonucleotide **20** (**20**-AuNP) the half-life was around 16 min (Table 3). The result obtained for **13**-AuNp is in good agreement with other gold nanoparticle conjugates functionalized with commercially available disulfide oligonucleotides [35]. Conjugation of AuNp with the 20-mer functionalized with the commercial 3'-thiol modifier gave similar results (supplementary information). Moreover, we found some discrepancies when we compared the stability results obtained for **20**-AuNp with other gold nanoparticle conjugates functionalized with commercially available alkanethiol oligonucleotides [34,35]. In our opinion, these discrepancies may be attributed to methodological differences that we introduced to obtain our conjugates. In our case the saline concentration of the buffers we used was lower (we used 0.15 M NaCl instead of 0.30 M NaCl). The other aspect to consider is the compound we used for the disulfide reduction (we used TCEP instead of DTT). In any case, the gold nanoparticles resulting from the reaction with thiol-oligonucleotide **20** under our experimental conditions have higher stability to DTT solutions than the gold nanoparticles resulting from the reaction with disulfide oligonucleotide **13**.

The degree of functionalization of gold nanoparticles conjugates was investigated using the fluoresceine-labelled oligonucleotides **14** and **21** (disulfide and thiol oligonucleotide, respectively) following a method described by Mirkin *et* *al*. [35,36]. The resulting functionalized gold nanoparticles **14**-AuNp and **21**-AuNp were characterized by UV-vis spectroscopy (Figure S6A, Supplementary Information). After functionalization only a small shift in the surface plasmon band was observed (λ_max_ 519 to 522 for both conjugates). Aliquots of **14**-AuNp and **21-**AuNp were treated with a DDT solution at room temperature for two hours. The surface coverage was obtained from the fluorescent intensity of the released oligonucleotides. By measuring the concentration of oligonucleotides and gold nanoparticle conjugates of each sample (three times, two sets of samples), an average number of oligonucleotides per particle was calculated (Table 3). It was found that **14**-AuNp has less surface coverage (~37 pmol/cm^2^) than the thiol oligonucleotide-gold nanoparticle conjugate **21**-AuNp (~54 pmol/cm^2^). Conjugation of AuNp with the 20-mer functionalized with the commercial 3'-thiol modifier gave similar results (supplementary information). The results obtained are in the range of thiol oligonucleotides-gold nanoparticle conjugates commercially available described in the literature [32,33,35,36,37]. We registered as well the fluorescence spectra of a functionalized gold nanoparticle sample (**14**-AuNP) before treatment with DTT and the fluorescence intensity of the released oligonucleotides once the sample was treated with DTT (Figure S6B, Supporting Information). Comparing both spectra we observed that the fluorescence from the bounded oligonucleotides was quenched by more than 98% by the gold nanoparticles. 

**Table 3:** Half-lives and surface coverage data for oligonucleotide-gold nanoparticle conjugates.

Conjugate name	t_1/2_ (min)	Surface coverage	
		Strands/particle	pmol/cm^2^
**13**-AuNp	1.8	----	----
**20**-AuNp	14.5	----	----
**14**-AuNp	----	63.0 ± 4.1	37.4 ± 2.8
**21**-AuNp	----	97.4 ± 6.8	53.9 ± 5.5

Finally, hairpin oligonucleotide (**11**) carrying one disulfide group in the middle of the loop was deprotected with TCEP (55 °C, o.n.) and the resulting thiol-oligonucleotide was reacted with 10 nm citrate-stabilized AuNp. The resulting functionalized AuNps were characterized by UV-vis spectroscopy, showing similar properties to the AuNp obtained with 5'- or 3'-thiolated oligonucleotides (Supplementary Information). This result shows the usefulness of the new thiol reagent developed in this work allowing the functionalization of AuNp of hairpin oligonucleotides using the loop positions. These interesting functionalized nanoparticles can not be produced with commercially available reagents.

## 3. Experimental

### 3.1. General

All reagents were purchased from Sigma-Aldrich or Fluka and were used without further purification. Dry solvents were purchased as well from Sigma-Aldrich or Fluka and used as supplied. All the standard phosphoroamidites and ancillary reagents used for oligonucleotide synthesis were purchased from Applied Biosystems or Link Technologies. Flash column chromatography was carried out on silica gel SDS 0.063–0.2 mm/70–230 mesh.

^1^H- and ^13^C-NMR spectra were recorded at 25 °C on a Varian Mercury 400 MHz spectrometer and the ^31^P-NMR spectra were recorded on a Varian Inova 300 MHz spectrometer using CDCl_3_. Tetramethylsilane (TMS) was used as an internal reference (0 ppm) for ^1^H spectra and the residual signal of the solvent (77.16 ppm) for ^13^C and ^31^P. Chemical shifts are reported in part per million (ppm) in the δ scale, coupling constants in Hz and multiplicity as follows: s (singlet), d (doublet), t (triplet), q (quadruplet), m (multiplet), br (broad signal). HPLC separations were performed using a Waters 2695 Separations Module with a Waters 2998 Photodiode Array Detector. UV analyses and melting curves were performed using a Jasco V-650 instrument equipped with a thermoregulated cell holder. Fluorescent measures were performed using a Jasco FP-6200 spectrofluorometer as well equipped with a thermoregulated cell holder. Electrospray ionization mass spectra (ESI-MS) of compounds **1**–**3** and **6** were recorded on a Micromass ZQ instrument with single quadrupole detector coupled to a Waters Alliance 2696 HPLC. Matrix-assisted laser desorption ionization time-of-flight (MALDI-TOF) mass spectra of oligonucleotides were recorded either on a Voyager-DE^TM^ RP spectrometer (Applied Biosystems) or in a Perseptive Biosystems Voyager DE-RP instrument (Applied Biosystems) in negative mode by using 2,4,6-trihydroxyacetophenone (THAP)/ammonium citrate (1:1) (THAP-CA as matrix and additive respectively). The mass spectra analysis of oligonucleotide **12** was performed in the negative ion mode on Synapt G1 HDMS travelling wave ion mobility mass spectrometer (Waters-Micromass). The experiment was performed using a 384 well plate and introduced by automated chip-based nanoelectrospray using a Triversa NanoMate (Advion BioSciences).

*3-(tert-Butyldisulfanyl)propanoic Acid* (**1**). A solution of 3-mercaptopropanoic acid (0.34 mL, 3.9 mmol) and triethylamine (1.1 mL 7.8 mmol) in anhydrous dimethylformamide (DMF) (15 mL) was prepared under argon. Di-*tert*-butyl-1-(*tert*-butylthio)-1,2-hydrazinedicarboxylate (2.5 g 7.8 mmol) was dissolved in anhydrous DMF (10 mL) and added dropwise to the stirred solution. The reaction mixture was stirred at room temperature overnight, and the reaction was complete as judged by TLC. The solvent was evaporated under reduced pressure. In order to remove the remaining DMF, the residue was dissolved in toluene and evaporated under reduced pressure. The procedure was repeated three times. We used two different methodologies to purify the carboxylic acid. Method 1: The resulting crude was dissolved with 100 mL 10% aqueous solution of sodium hydroxide and washed three times with 50 mL of hexane. The aquerous solution was acidified with hydrochloric acid fuming 37% until pH = 2–3, and then was extracted with 50 mL of ethyl acetate (×3). The extracts were combined, dried over MgSO_4_ and concentrated to dryness under reduced pressure. The desired product (pale yellow oil) was obtained with 57% yield (0.36 g). Method 2: The product was purified by chromatography on silica gel. The column was packed with silica gel using a 1% triethylamine solution in ethyl acetate. The byproducts were eluted with a gradient of ethyl acetate to ethyl acetate/methanol 100:10. Then, the product was eluted using a 1% acid acetic solution in ethyl acetate/methanol 100:10. The pure fractions were combined and the solvent was eliminated under reduced pressure. The resulting residue was dissolved with 100 mL of ethyl acetate and the organic phase was washed with 50 mL of water and 50 mL of a saturated NaCl aqueous solution. The organic phase was dried over anhydrous magnesium sulphate and concentrated to dryness under reduced pressure. The desired product (pale yellow oil) was obtained with 34% yield (0.26 g). TLC (ethyl acetate) *R_f_* = 0.35; ^1^H-NMR, δ_H_ (CDCl_3_): 2.92 (t, *J* = 7 Hz, 2H), 2.78 (t, *J* = 7 Hz, 2H), 1.34 (s, 9H); ^13^C-NMR, δ_C_ (CDCl_3_): 178.20 (CO), 48.27 (C), 34.58 (CH_2_), 34.26 (CH_2_), 30.16 (CH_3_); ESI-MS *m/z* (negative mode) [M−H]^−^ = 193.04, (M = 194.04 g/mol calculated for C_7_H_14_O_2_S_2_).

*N*-[2-(tert-Butyldisulfanyl)ethylcarbonyl]-*L-threoninol* (**2**). A solution of compound **1** (300 mg, 1.54 mmol), L-threoninol (162 mg, 1.54 mmol), 1-ethyl-3-(3-dimethylaminopropyl)carbodiimide (EDCI) (413 mg, 2.31 mmol), hydroxybenzotriazole (HOBt) (313 mg, 2.31 mmol), and *N*,*N*-diisopropylethylamine (0.40 mL, 2.31 mmol) in anhydrous DMF (15 mL) was prepared under argon. After stirring the reaction mixture at room temperature overnight, the reaction was complete as judged by TLC and then concentrated to dryness under reduced pressure. In order to remove the remaining DMF, the residue was dissolved in toluene and concentrated to dryness under reduced pressure (×3). The residue was dissolved in 100 mL of dichloromethane (DCM) and the organic phase was washed with 50 mL 10% NaCO_3_H aqueous solution (×2) and 50 mL of a saturated NaCl aqueous solution. The organic phase was dried over anhydrous magnesium sulphate and concentrated to dryness under reduced pressure. The pure compound was obtained as a white solid (346 mg, 80% yield). TLC (ethyl acetate) R*_f_* = 0.19. ^1^H-NMR, δ_H_ (CDCl_3_): 6.41 (d, *J* = 6.8 Hz, 1H), 4.19 (qd, *J* = 6.4 and 1.6 Hz, 1H), 3.88–3.82 (m, 3H), 2.98 (t, *J* = 7.0 Hz, 2H), 2.66 (t, *J* = 7.0 Hz, 2H), 1.34 (s, 9H), 1.23 (d, *J* = 6.4 Hz, 3H). ^13^C-NMR, δ_C_ (CDCl_3_): 172.23 (CO), 68.72 (CH), 64.86 (CH_2_), 55.12 (CH), 48.47 (C), 36.25 (CH_2_), 35.82 (CH_2_), 30.18 (CH_3_), 20.71 (CH_3_). ESI-MS *m/z* (positive mode) [M+H]^+^ = 282.13, (M = 281.11 g/mol calculated for C_11_H_23_NO_3_S_2_).

*O^1^-(4,4'-Dimethoxytriphenylmethyl)-N*-[2-(tert-butyldisulfanyl)ethylcarbonyl]-*L-threoninol* (**3**). Compound **2** (242 mg, 0.86 mmol) was dried by evaporation of anhydrous acetonitrile (ACN) under reduced pressure and then dissolved in anhydrous pyridine (10 mL) under argon. 4,4'-Dimethoxytriphenylmethyl chloride (320 mg, 0.94 mmol) was added with stirring and exclusion of moisture, and the reaction was allowed to proceed at room temperature overnight. Then the reaction was quenched with methanol (0.5 mL). The solvent was removed under reduced pressure, and the residue was dissolved in DCM (75 mL). The organic phase was washed with 5% aqueous NaCO_3_H solution (30 mL) and with saturated NaCl solution (30 mL). The organic phase was dried over anhydrous magnesium sulphate and filtered. After filtration and removal of the solvent under reduced pressure, the product was purified by chromatography on silica gel. The column was packed with silica gel using a 1% triethylamine solution in ethyl acetate/hexane 1:1. The product was eluted with a gradient of hexane/ethyl acetate (1:1) to 100% ethyl acetate. The pure compound was obtained as white foam (309 mg, 62%). TLC (ethyl acetate/hexane 2:1) R*_f_* = 0.35. ^1^H-NMR, δ_H_ (CDCl_3_): 7.39–6.83 (m, 13H), 6.16 (d, *J* = 8.8 Hz, 1H), 4.07 (qd, *J* = 6.4 Hz and 1.6 Hz, 1H), 3.95–3.91 (m, 1H), 3.79 (s, 6H), 3.44 (dd, *J* = 9.6 Hz and 4.2 Hz, 1H), 3.28 (dd, *J* = 9.6 Hz and 3.2 Hz, 1H), 2.99–2.95 (m, 2H), 2.64–2.61 (m, 2H), 1.34 (s, 9H), 1.13 (d, *J* = 6.4 Hz, 3H). ^13^C-NMR, δ_C_ (CDCl_3_): 171.39 (CO), 158.87 (CH), 158.86 (CH), 144.54 (C), 135.69 (C), 135.50 (C), 130.14 (CH), 128.26 (CH), 128.15 (CH), 127.26 (C), 113.56 (CH), 87.07 (C), 68.91 (CH), 65.52 (CH_2_), 55.46 (CH_3_), 53.64 (CH), 48.33 (C), 36.33 (CH_2_), 35.73 (CH_2_), 30.20 (CH_3_), 20.13 (CH_3_). ESI-MS *m/z* (negative mode) [M−H]^−^ = 582.24, (M = 583.24 g/mol calculated for C_32_H_41_NO_5_S_2_).

### 3.2. Synthesis of CPG Support *5* Functionalized with Compound *3*

Compound **3** was incorporated on a long-chain alkylamine-controlled pore glass support (LCAA-CPG) following the standard methodology using the hemisuccinate derivative **4**. Compound **3** (50 mg, 0.09 mmol) was dried by evaporation of anhydrous ACN under reduced pressure. Then, compound **3** was dissolved in anhydrous pyridine (5 mL) under argon. Succinic anhydride (21 mg, 0.21 mmol) and 4-dimethylaminopyridine (DMAP) (6 mg, 0.05 mmol) were added to the solution. After four hours of magnetic stirring at room temperature, the reaction was complete as judged by TLC (5% methanol in ethyl acetate, R*_f_* = 0.10). The solvent was removed under reduced pressure and the residue was dissolved in dichloromethane (20 mL). The solution was washed with saturated aqueous sodium chloride (15 mL). After drying the organic phase with magnesium sulphate and filtered, the solvent was evaporated under reduced pressure. The monosuccinate derivative **4**, which was used in the next step without further purification, was obtained as a white foam.

Commercial LCAA-CPG (CPG Inc., Lincoln Park , NJ, USA, 300 mg, 73 µmol amino/g) was placed into a polypropylene syringe fitted with a polypropylene disc and washed sequentially with DMF, methanol, THF, DCM, and ACN. Then, a solution of *O*^1^-(4,4'-dimethoxytriphenylmethyl)-*N*-[2-(tert-butyldisulfanyl)ethyl-carbonyl]-*O*^3^-(succinyl)-L-threoninol (**4**) (28 mg 0.045 mmol) and triethylamine (26 µL, 0.36 mmol) in 0.5 mL of anhydrous ACN was prepared. The solution was added to the resin and then a solution of 2-(1*H*-benzotriazole-1-yl)-1,1,3,3-tetramethyluronium tetrafluoroborate (TBTU) (59 mg 0.36 mmol) in 0.3 mL of anhydrous ACN was incorporated to the mixture and left to react for 30 min. The resin was washed with DMF, methanol, DCM and ACN. The incorporation of compound **4** was determined by DMT quantification. The coupling procedure was repeated once more time and the functionality of the resin was determined by DMT quantification (*f* = 22 µmol/g). Then, the resin treated with 500 µL of Ac_2_O/DMF 1:1 to cap free amino groups.

O^1^-(4,4'-Dimethoxytriphenylmethyl)-N-[2-(tertbutyldisulfanyl)ethyl-carbonyl]-O3-[2-cyanoethyl-N,N-diisopropylaminophosphinyl]-L-threoninol (**6**). Compound **3** (180 mg 0.31 mmol) was dried by evaporation of anhydrous ACN under reduced pressure. Then, compound **3** and N,N-diisopropylethylamine (215 µL, 1.23 mmol) were dissolved in anhydrous DCM (10 mL) under argon. The solution was cooled on ice and 2-cyanoethoxy-N,N'-diisopropylaminochlorophosphine (112 µL, 0.46 mmol) was added dropwise with a syringe. Afterward, the solution was stirred at room temperature for 1 h and 30 min. After this time the reaction was complete as judged by TLC. Then, 10 mL of DCM were added and the organic layer was washed with 5% NaCO_3_H aqueous solution (20 mL) and with saturated NaCl aqueous solution (20 mL). The organic phase was dried over magnesium sulphate and filtered, and the solvent was evaporated under reduced pressure. The residue was dissolved in a small amount of ethyl acetate/hexane 1:1 and purified by chromatography on silica gel. The column was packed with silica gel using a 5% triethylamine solution in ethyl acetate/hexane 1:1. The product was eluted with ethyl acetate/hexane 1:1. The pure compound was obtained as pale yellow foam (123 mg 56%). TLC (ethyl acetate/hexane 1:1) R_f_ = 0.48 and 0.38. ^31^P-NMR, δ_P_ (CDCl_3_, 81 MHz): 148.13 and 147.93, two isomers. ^1^H-NMR, δ_H_ (CDCl_3_): 7.42–6.80 (m, 13H), 5.88 and 5.72 (2d, J = 8.8 Hz and 9.2 Hz, respectively, two isomers, 1H), 4.39–4.31 (m, 1H), 4.25–4.11 (m, 1H), 3.79 and 3.78 (2s, two isomers, 6H), 3.57–3.46 (m, 2H), 3.26–3.08 (m, 2H), 3.02–2.90 (m, 2H), 2.60–2.55 (m, 4H), 2.41–2.36 (m, 2H), 1.32 (s, 9H), 1.25–0.99 (m, 15H). ^13^C-NMR, δ_C_ (CDCl_3_): 170.96 (C), 158.67 (CH), 145.07 and 144.99 (C, two isomers), 136.34 (C), 136.25 and 136.24 (C, two isomers), 130.37 (CH), 130.32 and 130.27 (CH, two isomers), 128.50 and 128.41 (CH, two isomers), 128.00 (CH), 127.00 and 126.96 (C, two isomers), 118.04 (C), 113.33 (CH), 113.30 and 113.28 (CH, two isomers), 86.37 and 86.29 (C, two isomers), 69.73 and 68.96 (CH, 2d, J = 14.4 and 16.4 Hz respectively, two isomers), 63.16 and 62.84 (CH_2_, two isomers), 58.48 and 58.09 (CH_2_, 2d, J = 19.5 and 19.5 Hz, respectively, two isomers), 55.44 and 55.41 (CH_3_, two isomers), 54.46 and 54.16 (CH, 2d, J = 4.9 and 6.3 Hz, respectively, two isomers), 48.25 and 48.21 (C, two isomers), 43.37 and 43.29 (CH, 2d, J = 12.3 and 12.5 Hz, respectively, two isomers), 36.51 and 36.43 (CH_2_, two isomers), 35.90 and 35.87 (CH_2_, two isomers), 30.17 (CH_3_), 24.93 and 24.91 (CH_3_, 2d, J = 7.7 and 7.8 Hz, respectively, two isomers), 24.76 and 24.57 (CH_3_, 2d, J = 6.8 and 7.1 Hz, respectively, two isomers), 20.66 and 20.53 (CH_3_, 2d, J = 6.9 and 6.7 Hz, respectively, two isomers), 19.85–19.79 (CH_2_, m, two isomers). ESI-MS m/z (positive mode) [M+Na]^+^ = 806.34, (M = 783.35 g/mol calculated for C_41_H_58_N_3_O_6_PS_2_).

### 3.3. Synthesis of Oligonucleotides

The unmodified oligonucleotides were synthesized on a DNA synthesizer (Applied Biosystems 3400) using 200-nmol scale LV200^®^ polystyrene supports and commercially available chemicals. The benzoyl (Bz) group was used for the protection of the amino group of C and A, and the isobutyryl (^i^Bu) group for the protection of G. The coupling yields were >97%. The last DMT group was removed at the end of the synthesis. Each solid support was treated with aqueous concentrated ammonia at 55 °C for 12 h to cleave the products from the supports and remove the benzoyl and isobutyryl groups. The mixtures were filtered and ammonia solutions were concentrated to dryness. Unmodified oligonucleotides were desalted with Sephadex G-25 (NAP-10 column) G-25 (NAP-10column) and analyzed by HPLC. Column: XBridge^TM^ OST C18 (4.6 × 50 mm, 2.5 µm). Solvent A: 5% ACN in 100 mM triethylammonium acetate (TEAA) (pH = 7) and solvent B: 70% ACN in 100 mM TEAA (pH = 7). Flow rate: 1 mL/min. Conditions: 10 min of linear gradient from 0 to 30% B. The resulting oligomers were analyzed by mass spectrometry (MALDI-TOF) and UV/Vis spectroscopy (data not shown) and used without further purification. The yields obtained ranged from 72 to 87%.

The oligonucleotide sequences containing the threoninol derivative at the 3'-end (**7**, **8**, **13** and **14**) were synthesized on a 0.5 µmol scale using the solid support **5**. Oligonucleotide sequence **7** was prepared using a *tert*-butyl hydroperoxide or the standard 0.02 M iodine solution for the oxidation of phosphites. Because better results were obtained using the standard 0.02 M iodine solution, all sequences were prepared in that way. The last DMT group was removed at the end of the synthesis. Each solid support was treated with aqueous concentrated ammonia at 55 °C for 12 h to cleave the products from the supports and remove the benzoyl and isobutyryl groups, except the support coming form the synthesis of oligonucleotide **7** that was treated with aqueous concentrated ammonia at room temperature for 1 h due to the absence of base protecting groups. Work-up was similar as described above. Oligonucleotide **7** (synthesis performed with iodine solution) was used without further purification after it was desalted with Sephadex G-25 (NAP-10 column), (74% yield, 36.2 OD_260_). Compound **8**, **13** and **14** were purified by HPLC on a Nucleosil 120C18 (10 μm, 200 × 10 mm) column. Solvent A: 5% ACN in 100 mM of TEAA (pH = 7) and solvent B: 70% ACN in 100 mM TEAA (pH = 7). Flow rate: 3 mL/min. Conditions: 20 min linear gradient from 0–50% B (oligonucleotides **8** and **13**), 20 min linear gradient from 15–38% B (oligonucleotide **14**). The resulting products were desalted with Sephadex G-25 (NAP-10 column) and analyzed by HPLC. Column: XBridge^TM^ OST C_18_ (4.6 × 50 mm, 2.5 μm). Solvent A: 5% ACN in 100 mM TEAA (pH = 7) and solvent B: 70% ACN in 100 mM TEAA (pH = 7). Flow rate: 1 mL/min. Conditions: 10 min linear gradient from 0–30% B, then 5 min. linear gradient from 30–100% B (oligonucleotides **7**, **8** and **13**), 4 min linear gradient from 0–12% B, then 1 min linear gradient from 12–40% B, then 5 min linear gradient from 40–45% B, then 5 min linear gradient from 45–100% B (oligonucleotide **14**). After HPLC purification the yields obtained ranged from 32 to 48%.

The oligonucleotide sequence **9** (threoninol modification at the 5'-end) and sequences **10**–**12** (threoninol modification in the middle), were synthesized on a 200 nmol scale employing LV200^®^ polystyrene supports as described above. Phosphoramidite **6** was used to incorporate the threoninol derivative at the 5'-end and in the middle of the corresponding oligonucleotides. The protected phosphoramidite was dissolved in anhydrous DCM instead of ACN to obtain a 0.1 M solution and was allowed to react for 300 s instead of 30 s used for an unmodified phosphoramidite. The DMT determination showed that the efficiency of coupling of the phosphoramidite was >90%. The last DMT group was removed at the end of the synthesis. Each resin was treated with aqueous concentrated ammonia at 55 °C for 12 h to cleave the products from the supports and remove the benzoyl and isobutyryl groups. In the case of oligonucleotide **12** only a small amount of the solid support was treated with ammonia for characterization purposes. Work-up was similar as described above. Oligonucleotide **9** was used without further purification after once desalted with Sephadex G-25 (NAP-10 column), (82% yield, 18.7 OD_260_). Compound **10**–**12** were purified by HPLC on a XBridge^TM^ OST C_18_ semipreparative column (10 × 50 mm, 2.5 μm). Solvent A: 5% ACN in 100 mM TEAA (pH = 7) and solvent B: 70% ACN in 100 mM TEAA (pH = 7). Flow rate: 3 mL/min. Conditions: 10 min linear gradient from 0–30% B, then 5 min linear gradient 30–100% B, at room temperature for oligonucleotide **10** and at 60 °C for oligonucleotides **11** and **12**. The resulting products were desalted with Sephadex G-25 (NAP-10 column) and analyzed by HPLC. Column: XBridge^TM^ OST C_18_ (4.6 × 50 mm, 2.5 μm) using the conditions described above except the flow rate that was 1 mL/min. All the purified oligonucleotides were analyzed as well by mass spectrometry. After HPLC purification 12.9 OD_260_ (50% yield) of compound **10** and 14.5 OD_260_ (42% yield) of compound **11** were obtained.

### 3.4. Removal of StBu Group and Conjugation of the Resulting Thiol Oligonucleotides

Oligonucleotides **7** and **8** (1–3 OD_260_) were dissolved in 300 μL of an aqueous solution 0.1 M TEAA (pH = 7). Then, 34 μL of an aqueous solution of 0.1 M TCEP were added to the solution and allowed to react at 55 °C. Different samples were analyzed by HPLC at different times of reaction (1 h, 3 h and 4 h) (Figure S4, Supplementary Information). Column: X-Bridge^TM^ OST C_18_ (2.5 μm 4.6 × 50 mm). Solvent A: 100 mM TEAA (pH = 7) and solvent B: 70% ACN in 100 mM TEAA (pH = 7). Flow rate: 1 mL/min. Conditions: 10 min linear gradient from 0–30% B, then 5 min linear gradient from 30–100% B. The oligonucleotide carrying a free thiol **15** was collected and analyzed by mass spectrometry (MALDI-TOF). The resulting thiol oligonucleotides **15** and **18** were purified with Sephadex G-10 (NAP-5 column). The oligonucleotide was eluted with 1 mL of sterile water. 

Conjugation with 2-bromo-2'-hydroxy-5'-nitroacetanilide: The solution from the NAP column was concentrated to 500 μL and 60 μL of an aqueous solution 1 M of hydrogen carbonate (pH = 9) were added. Then, 100 μL of a solution 2.25 mM of 2-bromo-2'-hydroxy-5'-nitroacetanilide in DMF were added and allowed to react at room temperature overnight. The reaction mixtures were desalted on a NAP-5 column. The DNA fractions were analyzed by HPLC. The main compounds were isolated and analyzed by mass spectrometry. Results are summarized in Table 1.

Conjugation with *N*-(1-pyrenyl)maleimide: The solution was concentrated to 500 μL and 60 μL of an aqueous solution 1 M TEAA (pH = 7) were added. Then, 100 μL of a solution 2.25 mM of *N*-(1-pyrenyl)maleimide in DMF were added and allowed to react at room temperature overnight. The reaction mixture was desalted on a NAP-5 column. The DNA fractions were purified by HPLC. The main compounds were isolated and analyzed by mass spectrometry and UV/Vis spectroscopy (λ_max_ = 256 and 342 nm). Results are summarized in Table 1.

### 3.5. Preparation of Oligonucleotide-Gold Nanoparticle Conjugates

Citrate stabilized gold nanoparticles (9.7 nm) were purchased from BBI Life Sciences and used as received. To prepare the conjugates, 5 nmol (1 OD_260_ unit) of disulfide modified-oligonucleotide (**13** and **14**) were added to 1 mL of the gold nanoparticle solution (9.4 nM). Disulphide oligonucleotides **13** and **14** were converted to their corresponding thiol oligonucleotides (**20** and **21** respectively) by reaction with TCEP and as well were used to functionalize gold nanoparticles. Then, 20 nmol (4 OD_260_) of each oligonucleotide were deprotected following the conditions described above. The resulting thiol oligonucleotides were desalted on a NAP-5 column. Freshly deprotected oligonucleotides (5 nmol, 250 μL) were added to a 1 mL of gold colloid. The mixture was shaken for 16–20 h prior to salt stabilization. Then the solution was brought to a final concentration of 10 mM sodium phosphate (pH = 7.2). The mixture was then allowed to equilibrate for 30 min before bringing the concentration to 0.15 M NaCl over a 7 h 30 min period in a stepwise manner (0.05 M NaCl increments each 2 h 30 min). The solutions were sonicated for 20 s. before each addition to keep the particles dispersed during the salting procedure. The salting process was followed by incubation overnight at room temperature. Finally, to remove all unbound oligonucleotides, the solution was centrifuged at 13,200 rpm (16,100 × *g*) for 30 min. The supernatant was removed and the reddish solid at the bottom of the centrifuge tube was dispersed in 1 mL of a 0.15 M, 10 mM sodium phosphate buffer (pH = 7.2). This procedure was repeated three times. Approximately 20–30% of the original nanoparticle concentration may be lost during centrifugation and work-up. As we wanted to compare stabilities of conjugates obtained with disulfide and thiol oligonucleotides (**13**-AuNp and **20**-AuNp) disulfide and thiol oligonucleotides we dispersed the reddish residue in 500 μL of 0.15 M NaCl, 10 mM sodium phosphate (pH = 7.2), 0.1% NaN_3_ solution. Then, each solution was analyzed by UV-visible absorption spectroscopy and the concentration of gold nanoparticle conjugates was adjusted to 7.0–7.2 nM by adding the required volume of buffer. The conjugates obtained with the fluorescent oligonucleotides (**14**-AunP and **21**-AuNp) were directly resuspended in 1 mL of a 0.15 M NaCl, 10 mM sodium phosphate (pH = 7.2), 0.1% NaN_3_ solution. The extinction coefficient used was the same as that used for unmodified nanoparticles (ε_520_ = 7.6 × 10^7^ M^−1^cm^−1^, data provided by the manufacturer). Each solution was then stored in the fridge (4 °C) prior to use.

### 3.6. Stability Tests of Oligonucleotide-Gold Nanoparticle Conjugates

The effect of the disulphide or thiol moieties on the stability of oligonucleotide-nanoparticle conjugates was assessed by treating the conjugates with DTT (10 mM final concentration) at 40 °C. UV-vis spectra were recorded for both thiolated conjugates (**13** and **20**) at 1 min intervals. To give an indication of the durability of the oligonucleotide conjugates, absorbance at 675 nm was monitored against time. Then, the half-life (time to reach half the value for complete aggregation) was calculated for each conjugate.

### 3.7. Measuring Oligonucleotide Loading on Gold Nanoparticles

To determine the number of oligonucleotides loaded on each particle, the concentration of nanoparticles and the concentration of fluorescent DNA in each sample were measured. The fluorescent oligonucleotides were displaced from the nanoparticle surface using DTT. Then 200 μL of the gold nanoparticle conjugate solution was treated with 200 μL of a 1 M DTT solution in 0.18 M sodium phosphate buffer, (pH = 8) at room temperature for two hours. The sample was centrifuged to remove the sediment from the supernatant and the fluorescence spectra was recorded (conditions: λ_Ex_ = 495 nm and λ_Em_ = 510–550 nm). For each gold nanoparticle conjugate five different aliquots were prepared. Standard curves were prepared with known concentrations of the fluorescein disulfide oligonucleotide **14** using the same buffer, salt and DTT concentrations were prepared. The fluorescents values when correlated with the standard calibration curve gave a concentration for the fluorescent oligonucleotide in solution. The average number of oligonucleotides per particle was obtained by dividing the measured oligonucleotide molar concentration by the original conjugate gold nanoparticle concentration. Normalized surface coverage values were calculated by dividing by the surface area of the sphere which can be easily converted to pmol/cm^2^.

### 3.8. Denaturation Studies

Oligonucleotide duplexes (Table 2, entries 1–6) were dissolved in 0.3 M NaCl, 10 mM sodium phosphate buffer (pH = 7.0). The melting experiments were performed in duplicate at 3.5 μM concentration of oligonucleotide. The samples were heated at 90 °C for 5 min, allowed to cool slowly to room temperature to induce annealing and then kept overnight in a refrigerator at 4 °C. The melting curves were recorded by heating the samples with a temperature controller from 5 to 75 °C at a constant rate of 1 °C/min and monitoring the absorbance at 260 nm. Denaturation curves for hairpin oligonucleotides **11** and the corresponding unmodified hairpin were performed in duplicate at 2.8 μM oligonucleotide concentration in a 50 mM NaCl, 10 mM sodium phosphate buffer (pH = 7.0) (entries 7 and 8). The melting curves were recorded by heating the samples with a temperature controller from 20 to 85 °C at a constant rate of 1 °C/min and monitoring the absorbance at 260 nm. During the experiment, when the temperature was below 25 °C, argon was flushed to prevent water condensation on the cuvettes. The melting experiments were recorded in 1 cm path-length cells. The T_m_ values were calculated with the *Meltwin 3.2* software [38].

## 4. Conclusions

In summary, the preparation of novel monomer units for the introduction of thiol groups in oligonucleotides is described. The phosphoramidite derivative and functionalized solid support are easily prepared and allow the introduction of thiol group at terminal and internal positions of oligonucleotides. The monomers are made from simple and commercially available building blocks: L-threoninol and 3-mercaptopropionic acid. Several oligonucleotides of different lengths carrying thiol groups were prepared and obtained in good yields demonstrating the utility of the novel monomer units. During the optimization of solid-phase synthesis protocols we observed that standard iodine solution is preferred to *tert*-butyl hydroperoxide solution and that ammonia treatment at 55 °C for extended time can be detrimental for the integrity of the novel thiol derivative. The use of mild protecting groups is recommended to minimize this last side reaction although the extension of the side reaction is not high. The resulting thiol-oligonucleotides react specifically and efficiently with molecules bearing maleimido- and bromoacetamido- groups and they can be used for the functionalization of gold nanoparticles. The new reagent allows the introduction of thiol group at any position of the sequence and, for this reason; it was possible to demonstrate the modification of gold nanoparticles through the reaction of the thiol group located in the middle of the oligonucleotide.

## Supplementary Materials

Supplementary materials can be accessed at: http://www.mdpi.com/1420-3049/17/9/10026/s1.
