# Supplementary material for: Synthesis of Oligonucleotides Carrying Thiol Groups Using a Simple Reagent Derived from Threoninol
Source: Molecules. 2012 Aug 24;17(9):10026–45. doi: 10.3390/molecules170910026 (PMC6268497; doi:10.3390/molecules170910026)

**Electronic Supplementary Information (ESI)**

**Table S1.** Unmodified oligonucleotides prepared in this study.

| # | Sequence (5'-3') | Description |
|---|---|---|
| **22** | CCTTGCCCTTTT | Complementary to **8** |
| **23** | AAAAGGGCAAGG | Unmodified version of **8** |
| **24** | TTTCCTGATAGT | Complementary to **10** |
| **25** | ACTATCAGGAAA | Unmodified version of **10** |
| **26** | CGGTAATTGGAA | Complementary to **9** |
| **27** | TTCCAATTACCG | Unmodified version of **9** |
| **28** | CTGTTGGCTTTTGCCAACAG | Unmodified hairpin version of **11** |

**Table S2.** Half-lives and surface coverage data for oligonucleotide-gold nanoparticle conjugates. Comparison of the data obtained with oligonucleotides carrying the threoninol derivative described in this work with the results obtained with oligonucleotides carrying the commercially available 3'-thiol modifier (DMT-O-$(CH_2)_3$-S-S-$(CH_2)_3$-O-succinate-CPG).

Oligonucleotide sequences:
**29**, CGGAGGTACATTCGACTTGA-**Y**;
**30**, Fluoresceine-CGGAGGTACATTCGACTTGA-**Y**;
**31**, CGGAGGTACATTCGACTTGA-**Z**;
**32**, Fluoresceine-CGGAGGTACATTCGACTTGA-**Z**;
being **Y** = -phosphate-$(CH_2)_3$-*S*-*S*-$(CH_2)_3$-OH; **Z** = -phosphate-$(CH_2)_3$-SH.

| Conjugate name | $t_{1/2}$ (min) | Surface coverage | |
|---|---|---|---|
| | | Strands/particle | pmol/cm$^2$ |
| **13**-AuNp | 1.8 | ---- | ---- |
| **20**-AuNp | 14.5 | ---- | ---- |
| **14**-AuNp | ---- | 63.0 ± 4.1 | 37.4 ± 2.8 |
| **21**-AuNp | ---- | 97.4 ± 6.8 | 53.9 ± 5.5 |
| **29**-AuNp | 2.1 | ---- | ---- |
| **31**-AuNp | 16.2 | ---- | ---- |
| **30**-AuNp | ---- | 49.7 ± 6.1 | 29.5 ± 4.2 |
| **32**-AuNp | ---- | 114.0 ± 6.7 | 63.1 ± 5.4 |

**Scheme S1.** Potential mechanisms to explain the formation of the side compound during extended ammonia treatment.

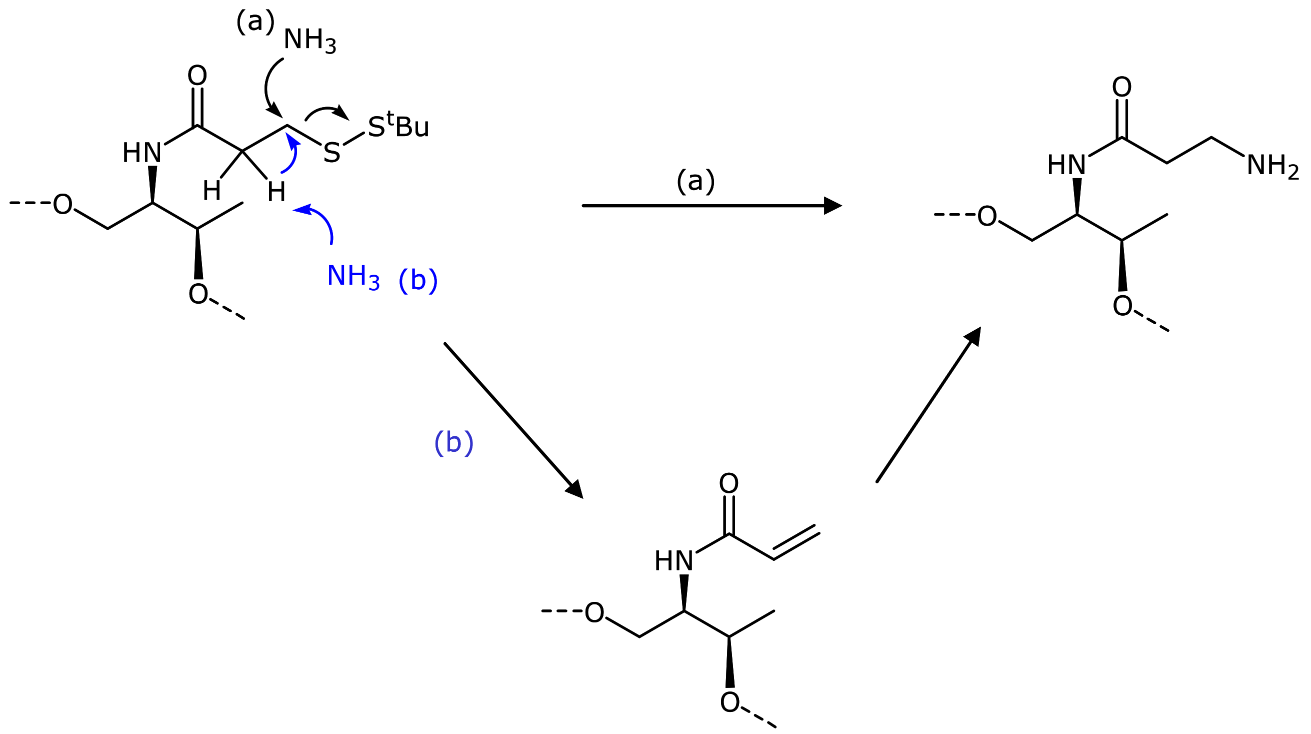

**Figure S1.** HPLC profiles of (**A**) oligonucleotide **9**, (**B**) oligonucleotide **10**, (**C**) oligonucleotide **8**, (**D**) oligonucleotide **11**, (**E**) oligonucleotide **12** before HPLC purification, (**F**) oligonucleotide **12** after HPLC purification. For HPLC profiles D, E and F the HPLC column was heated at 60 °C to avoid secondary structures.

**A)**

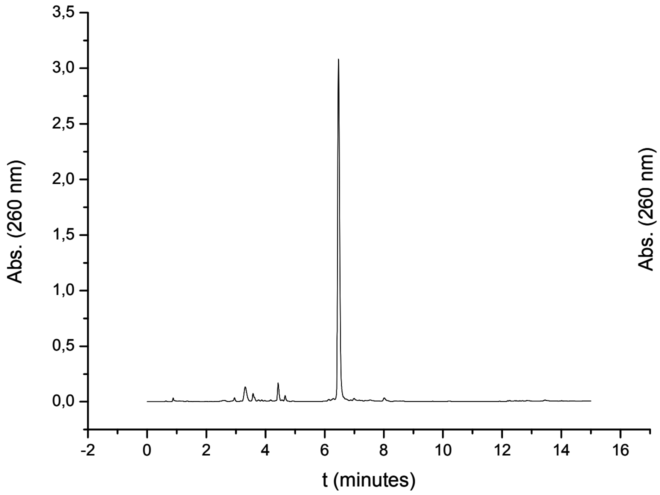


**B)**

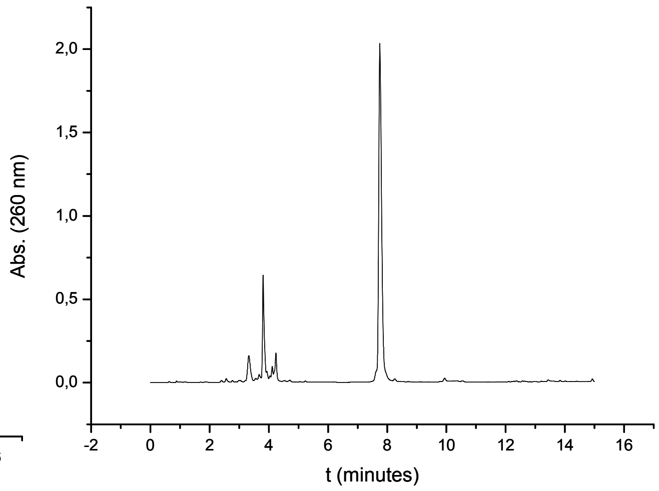


**C)**

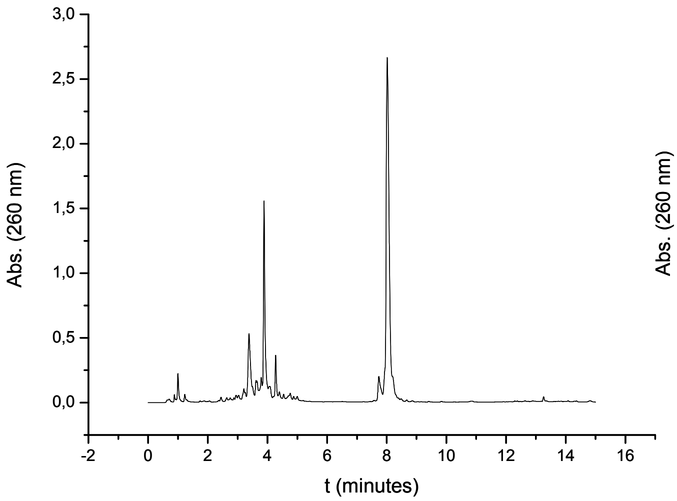


**D)**

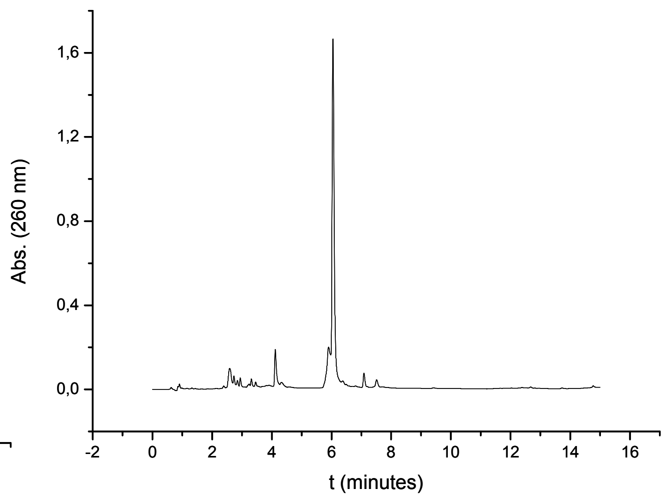


**E)**

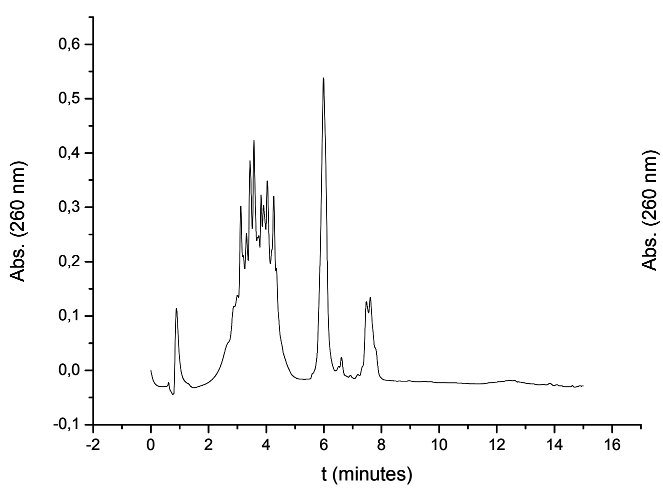


**F)**

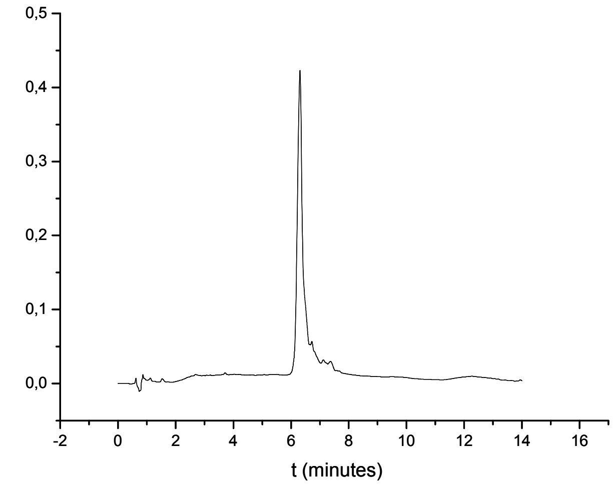

**Figure S2.** HPLC profiles before (below) and after (top) HPLC purification of (**A**) oligonucleotide **13**, (**B**) oligonucleotide **14**.

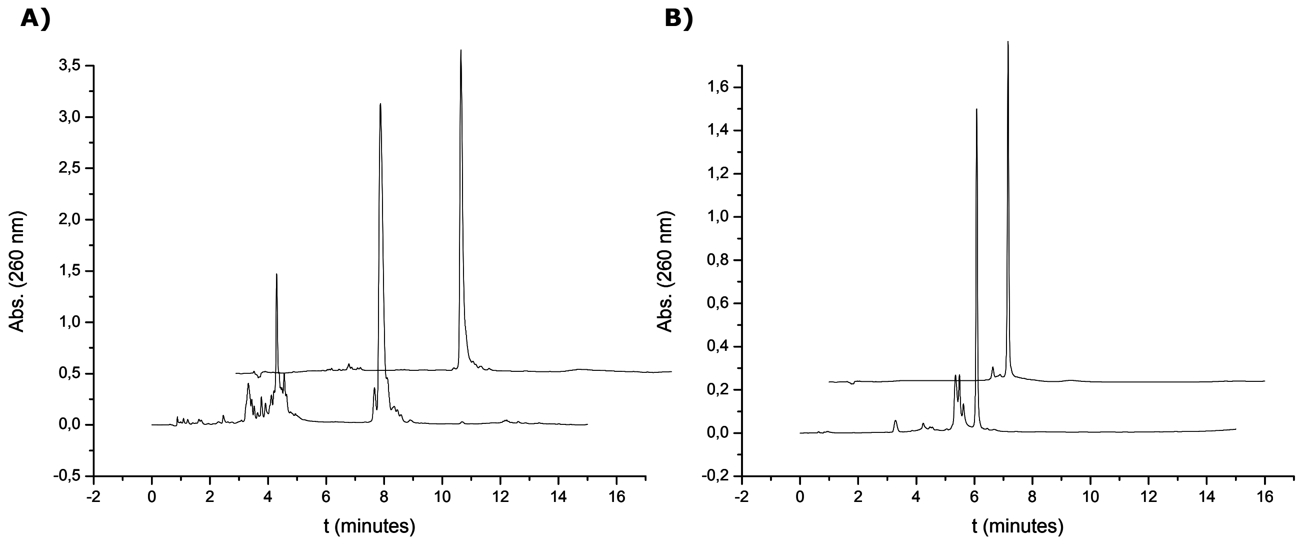


**Figure S3.** Melting curves of duplexes carrying *tert*-butylsulfanyl threoninol derivatives compared with unmodified duplexes. Blue: melting curve for the duplexes carrying *tert*-butylsulfanyl threoninol derivative. Black: melting curve for the corresponding unmodified duplex. (**A**) Oligonucleotide **8** and the corresponding complementary sequence. (**B**) Oligonucleotide **10**. (**C**) Oligonucleotide **9**. (**D**) Hairpin oligonucleotide **11**. Buffer conditions for (**A**), (**B**) and (**C**): 0.3M NaCl, 10 mM sodium phosphate buffer pH 7.0. (**D**) 50 mM NaCl, 10 mM sodium phosphate buffer pH 7.0.

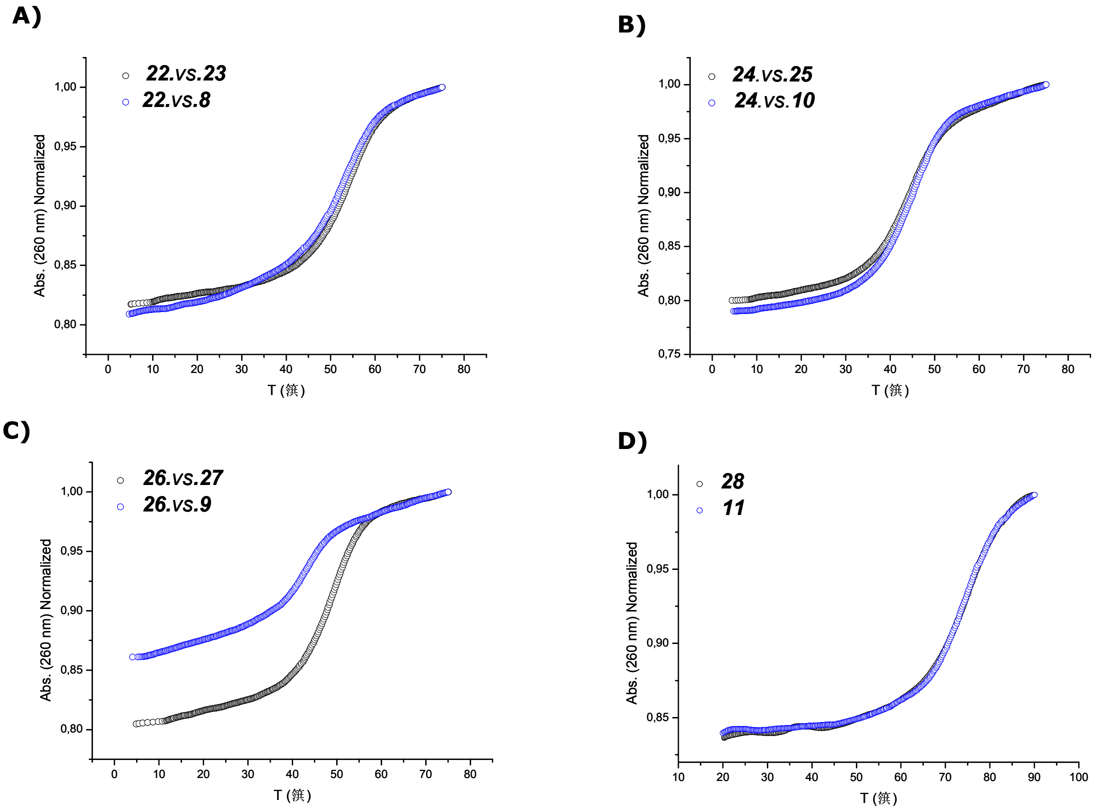

**Figure S4.** Removal of the StBu group followed by HPLC. HPLC profiles of (**A**) oligonucleotide **8**, (**B**) reaction mixture after treatment of oligonucleotide **8** with TCEP for 1 h, (**C**) 3 h and (**D**) 4 h.

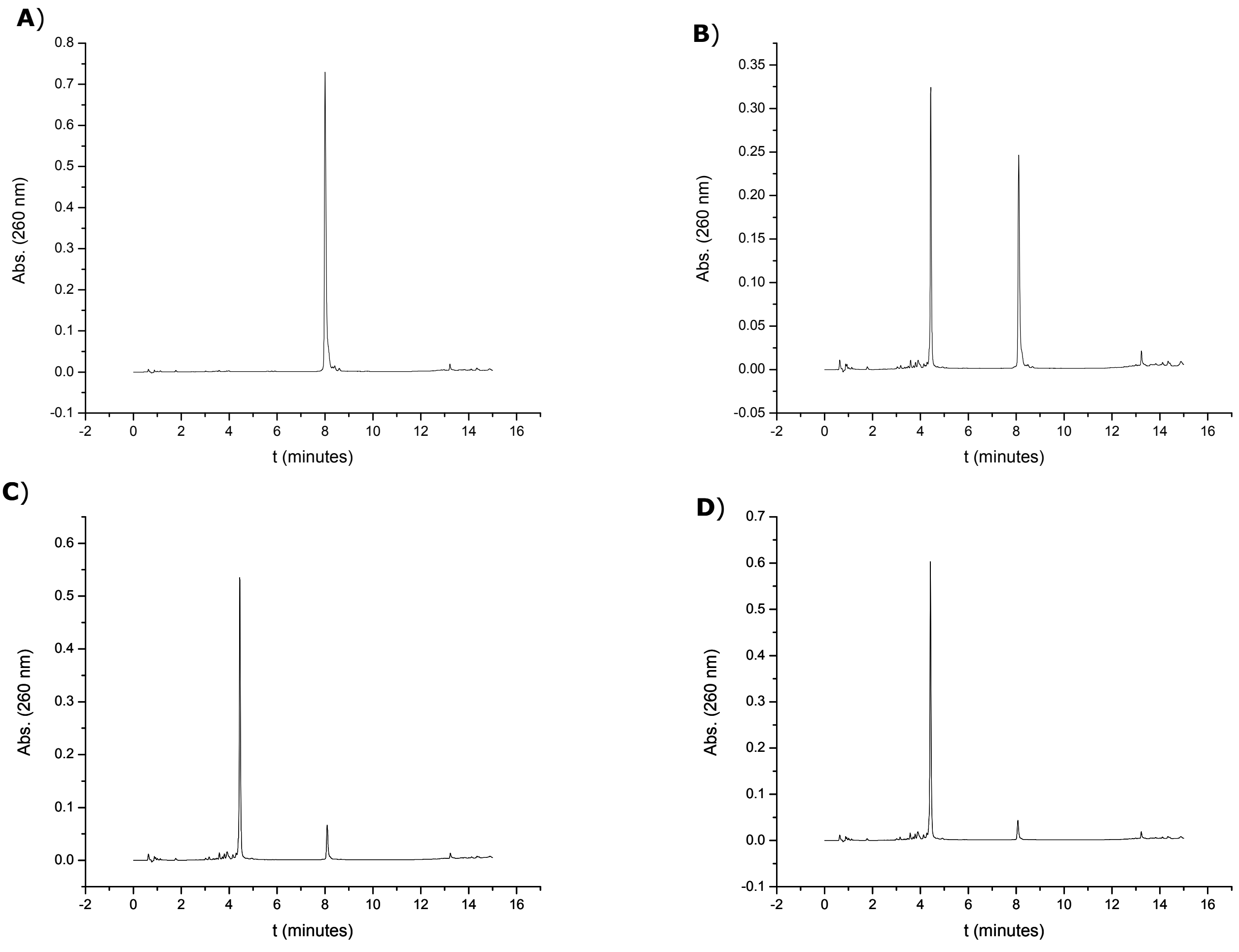


**Figure S5.** Conjugation of oligonucleotide **7** with 2-bromo-2'-hydroxy-5'-nitroacetanilide. HPLC profiles of (**A**) oligonucleotide **7**, (**B**) reaction mixture after treatment of oligonucleotide **7** with TCEP and (**C**) conjugation with 2-bromo-2'-hydroxy-5'-nitroacetanilide.

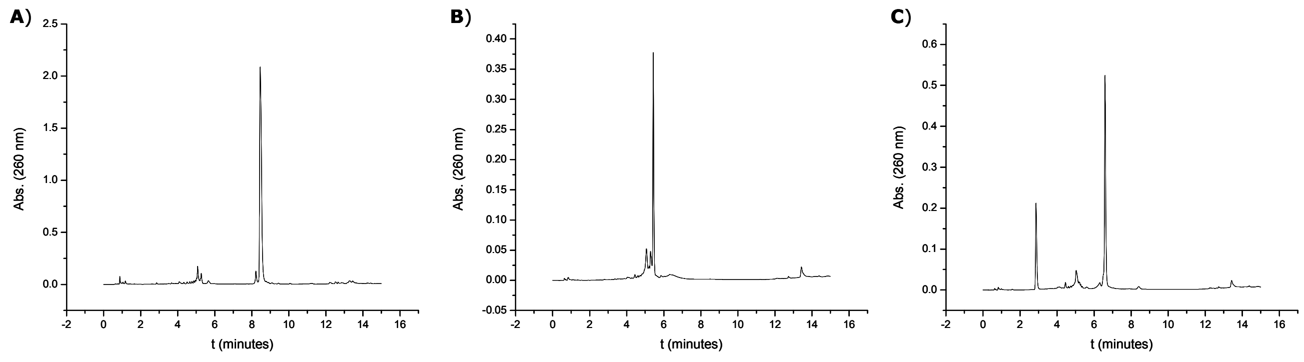

**Figure S6.** (**A**) UV-vis spectra of the initial gold nanoparticle solution and the functionalized gold nanoparticles with oligonucleotides **14** and **21**. (**B**) Fluorescence spectra of a **14**-AuNp sample (black line) and after treatment with DTT (blue line), ($\lambda_{Ex}$ = 493 nm).

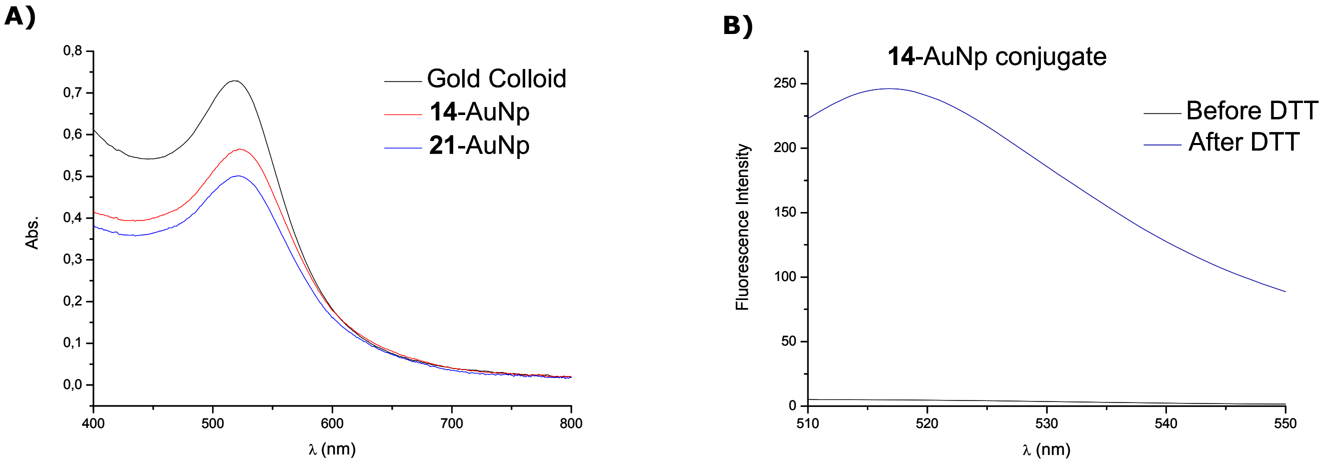


**Figure S7.** Time evolution of the absorbance changes monitored at 675 nm for **29**-AuNp and **31**-AuNp in 10 mM DTT.

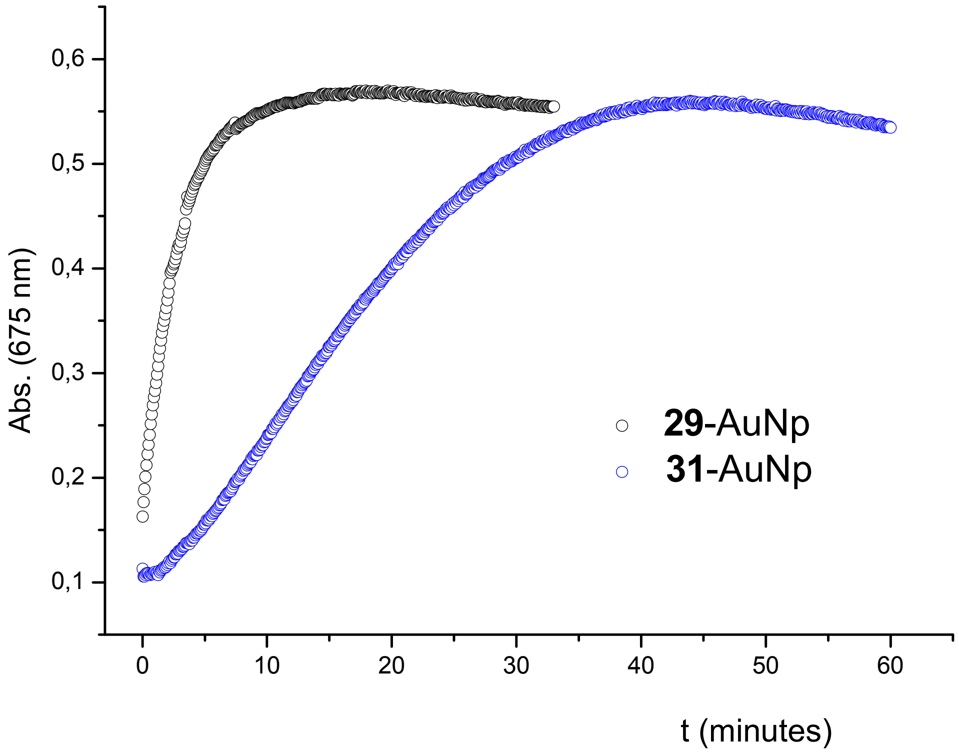

**Figure S8.** UV-vis spectra of the initial gold nanoparticle solution and the functionalized gold nanoparticles with hairpin oligonucleotide carrying the thiol threoninol derivative in the middle of the loop. Oligonucleotide sequence **11** was treated with TCEP (55 °C, o.n.) and the resulting thiol oligonucleotide (**33**) was reacted with 10 nm citrate-stabilized gold nanoparticles.

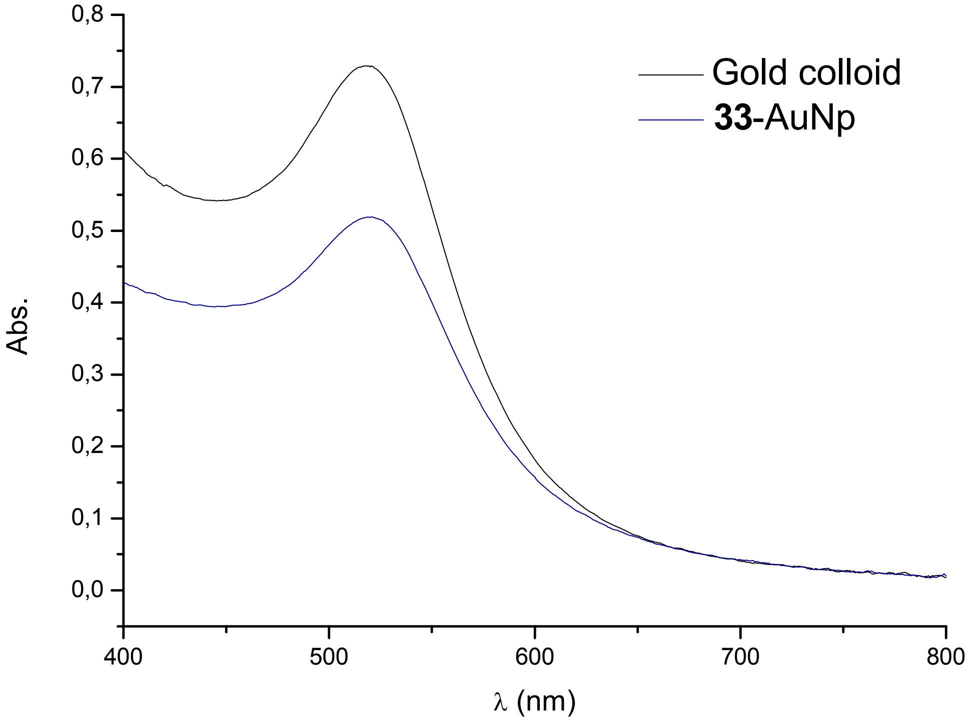

$^{1}$H-NMR spectrum of compound **1**.

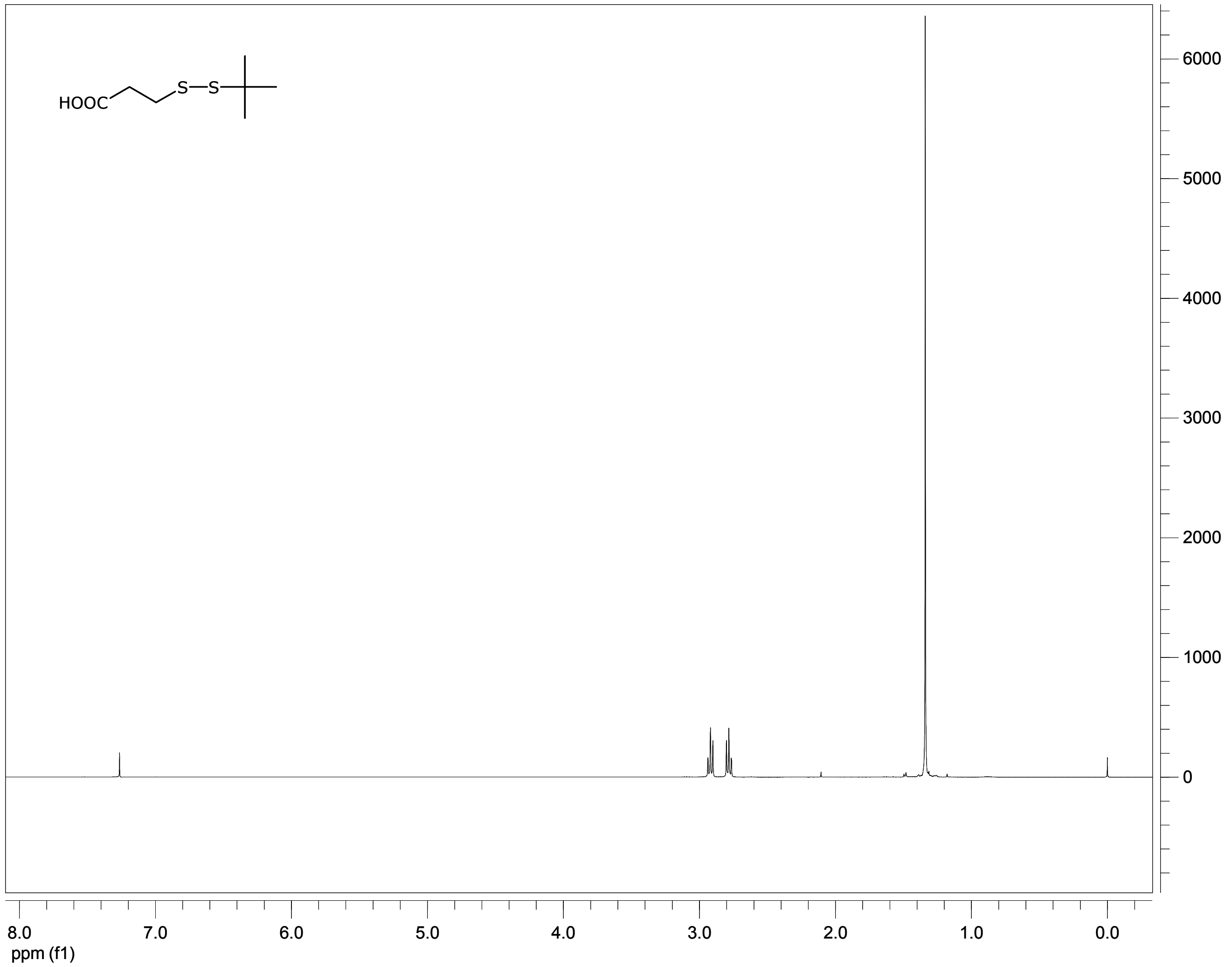

$^{13}$C-NMR spectrum of compound **1**.

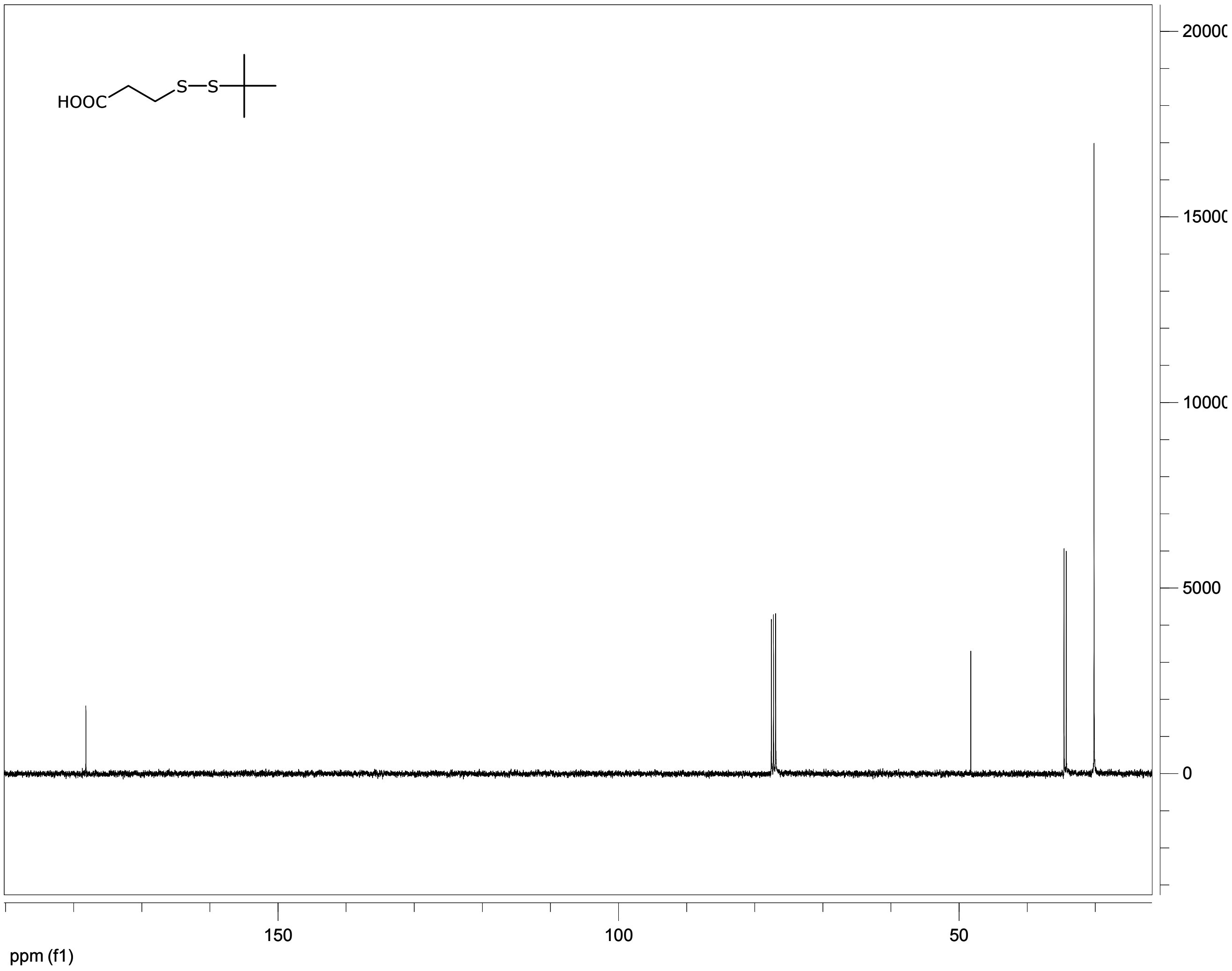

$^{1}$H-NMR spectrum of compound **2**.

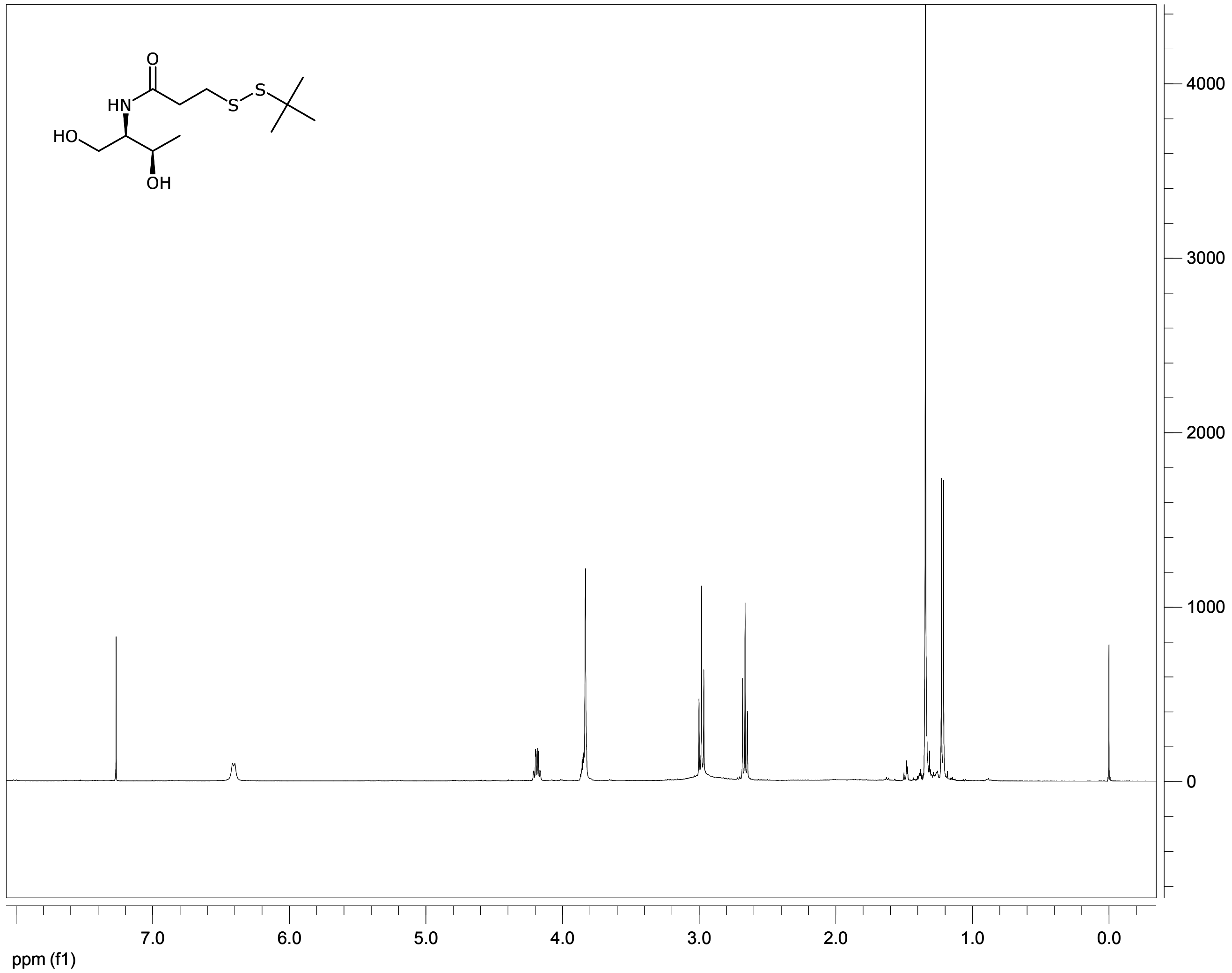

$^{13}$C-NMR spectrum of compound **2**.

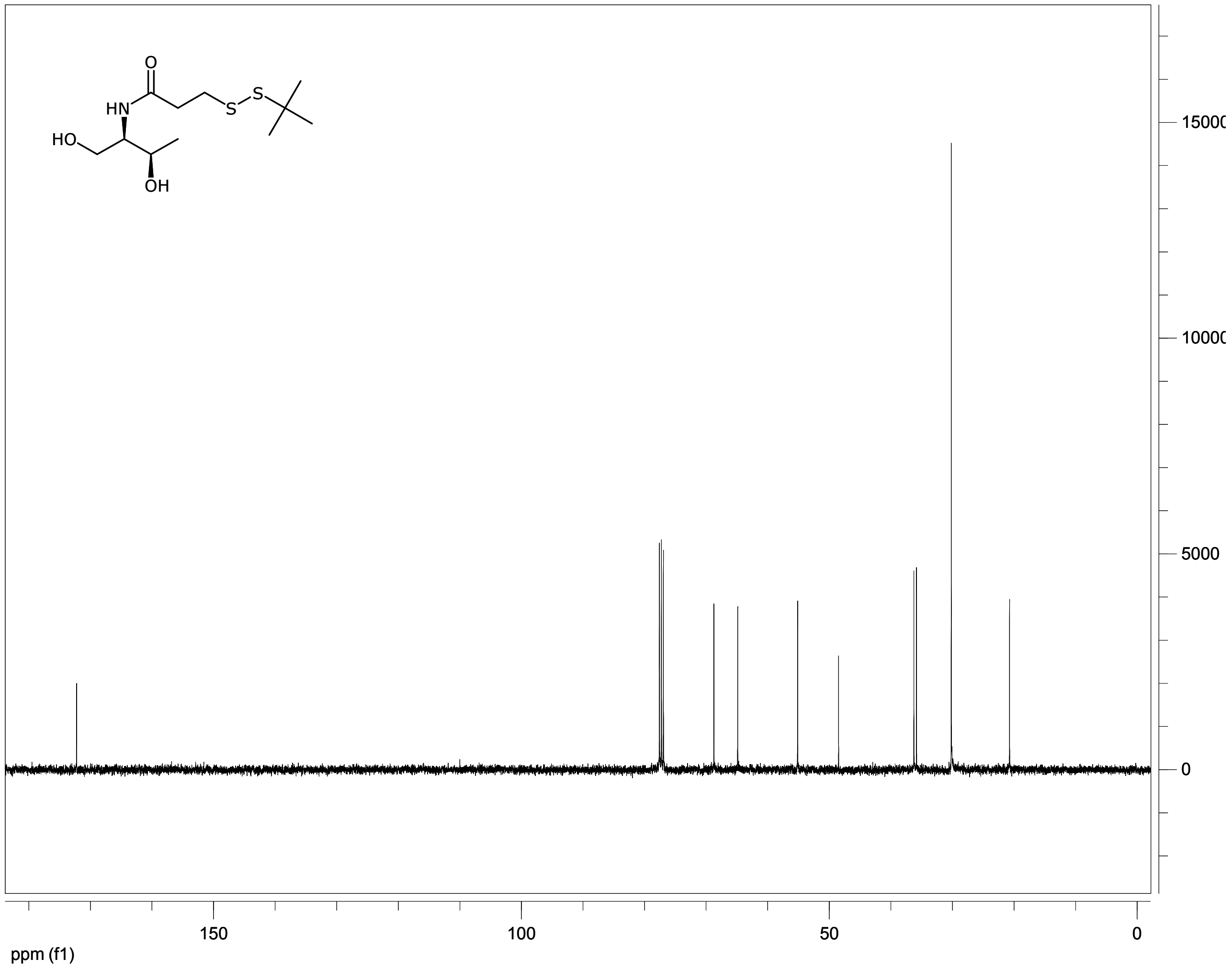

$^{1}$H-NMR spectrum of compound **3**.

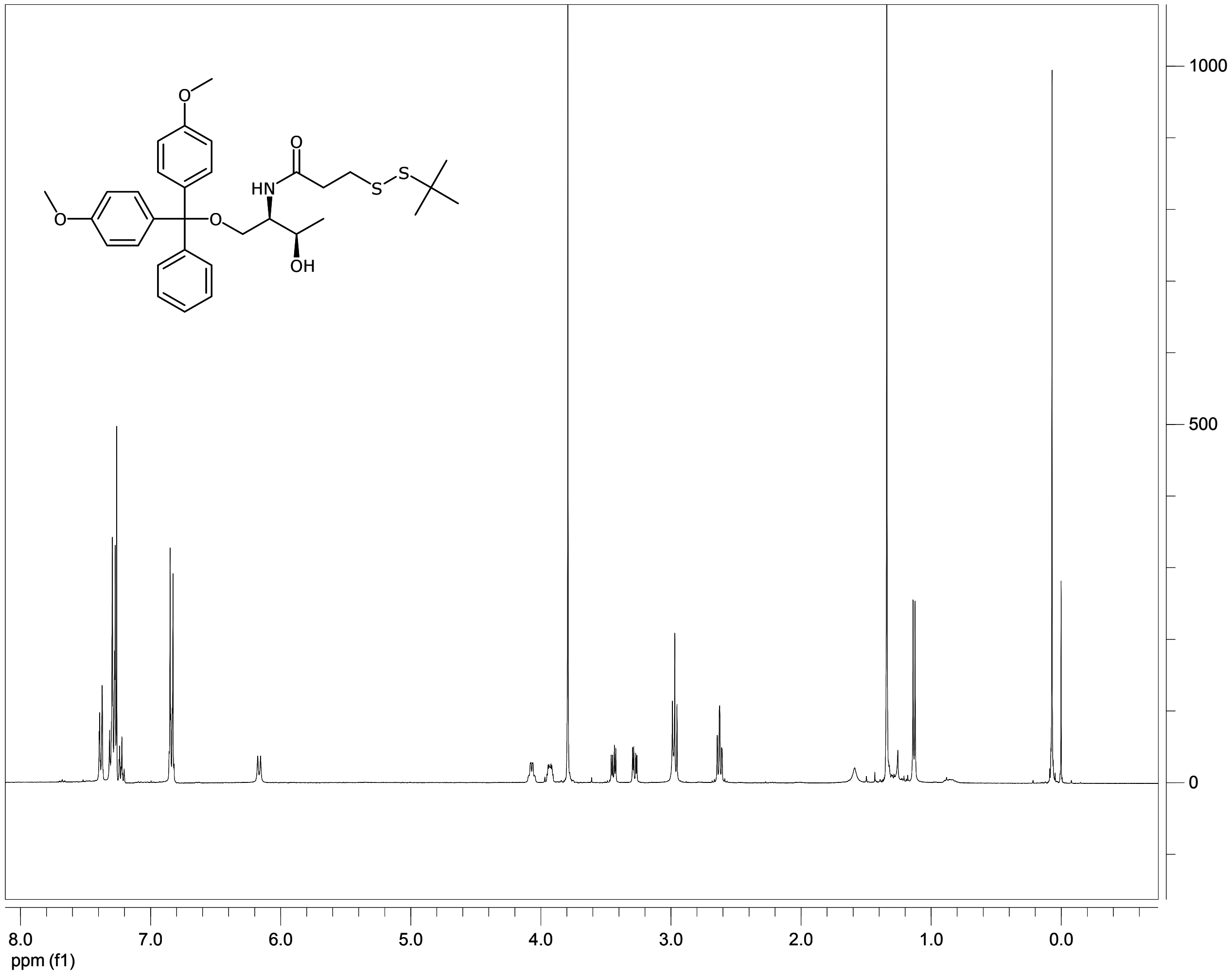

$^{13}$C-NMR spectrum of compound **3**.

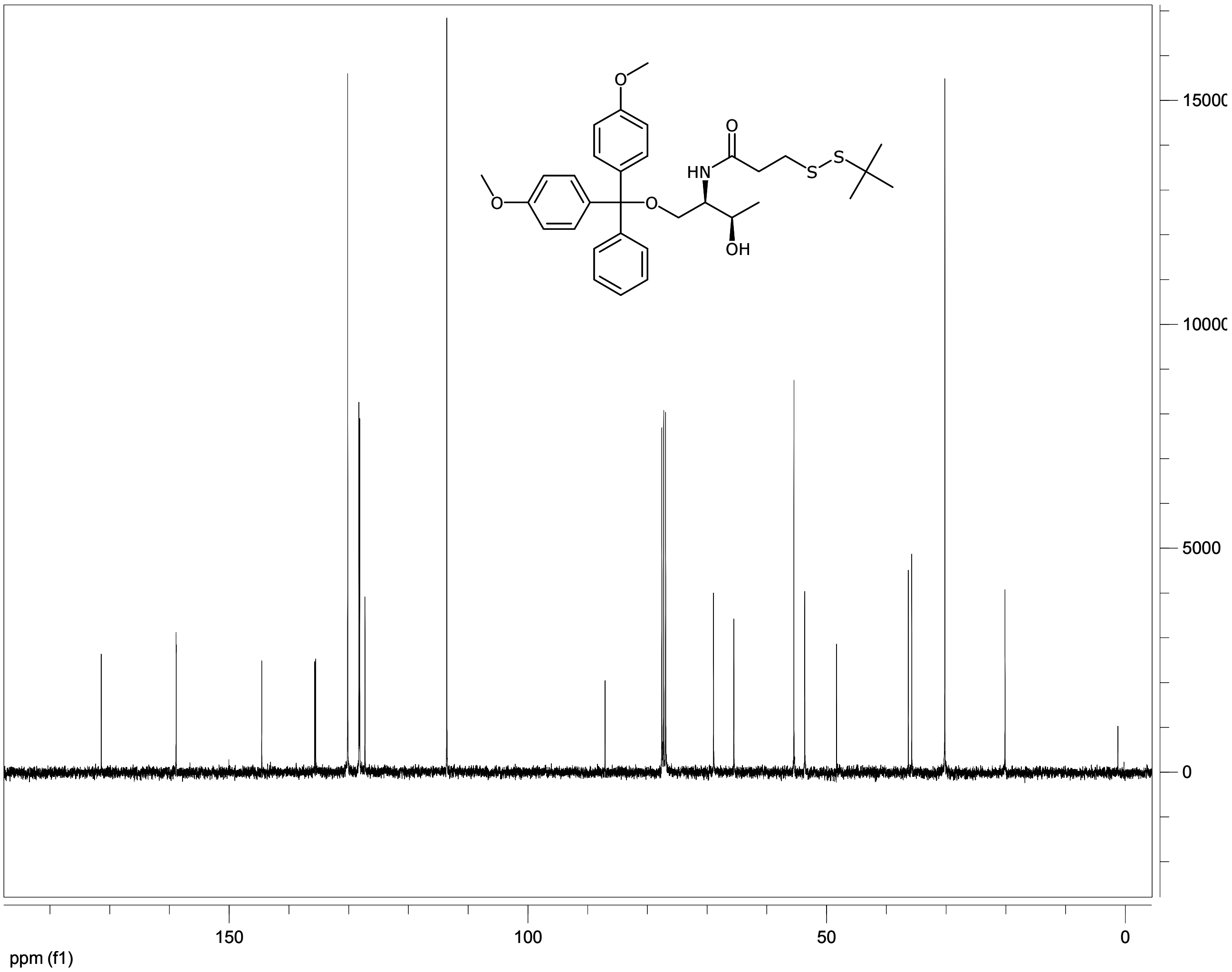

$^{1}$H-NMR spectrum of compound **6**.

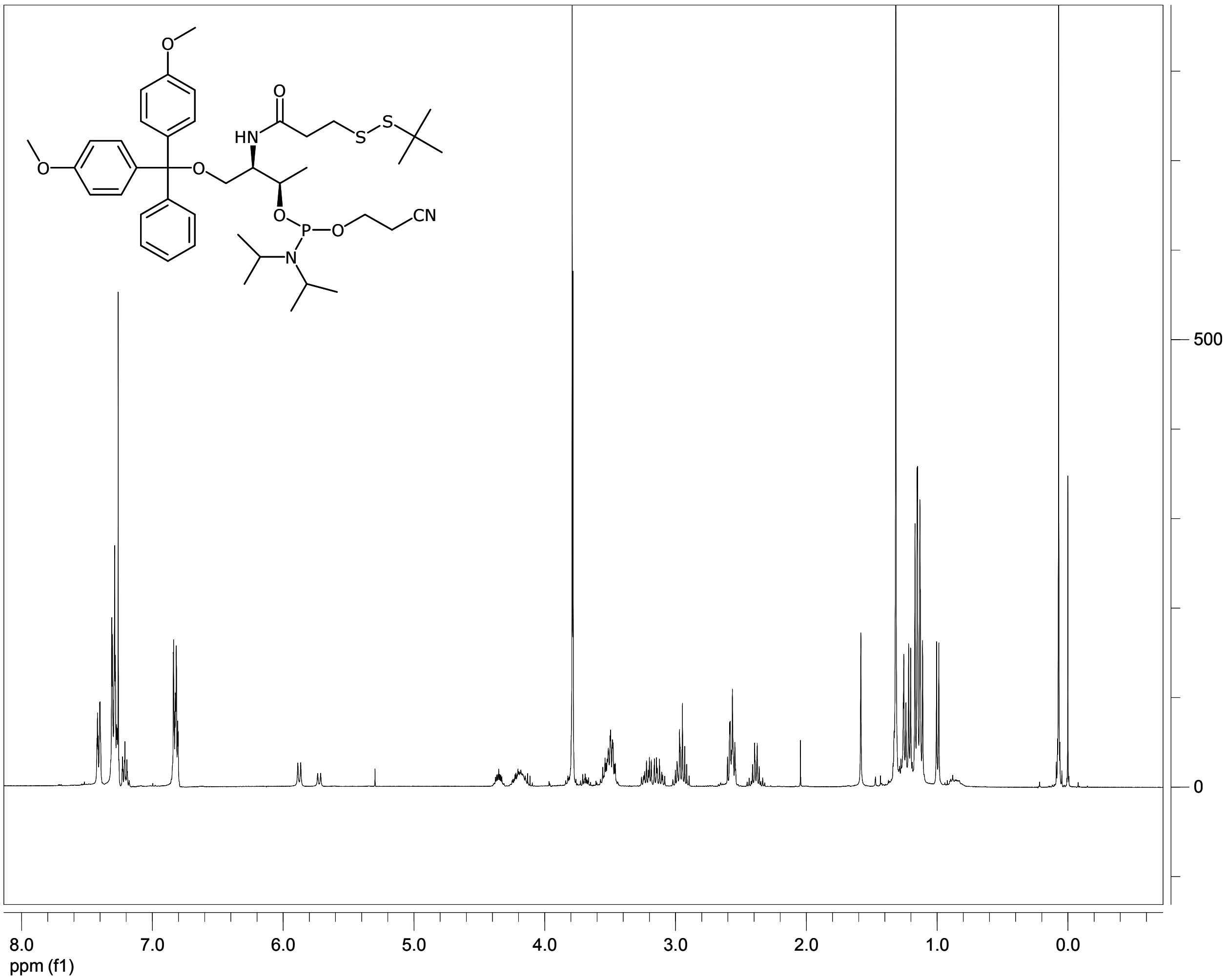

$^{13}$C-NMR spectrum of compound **6**.

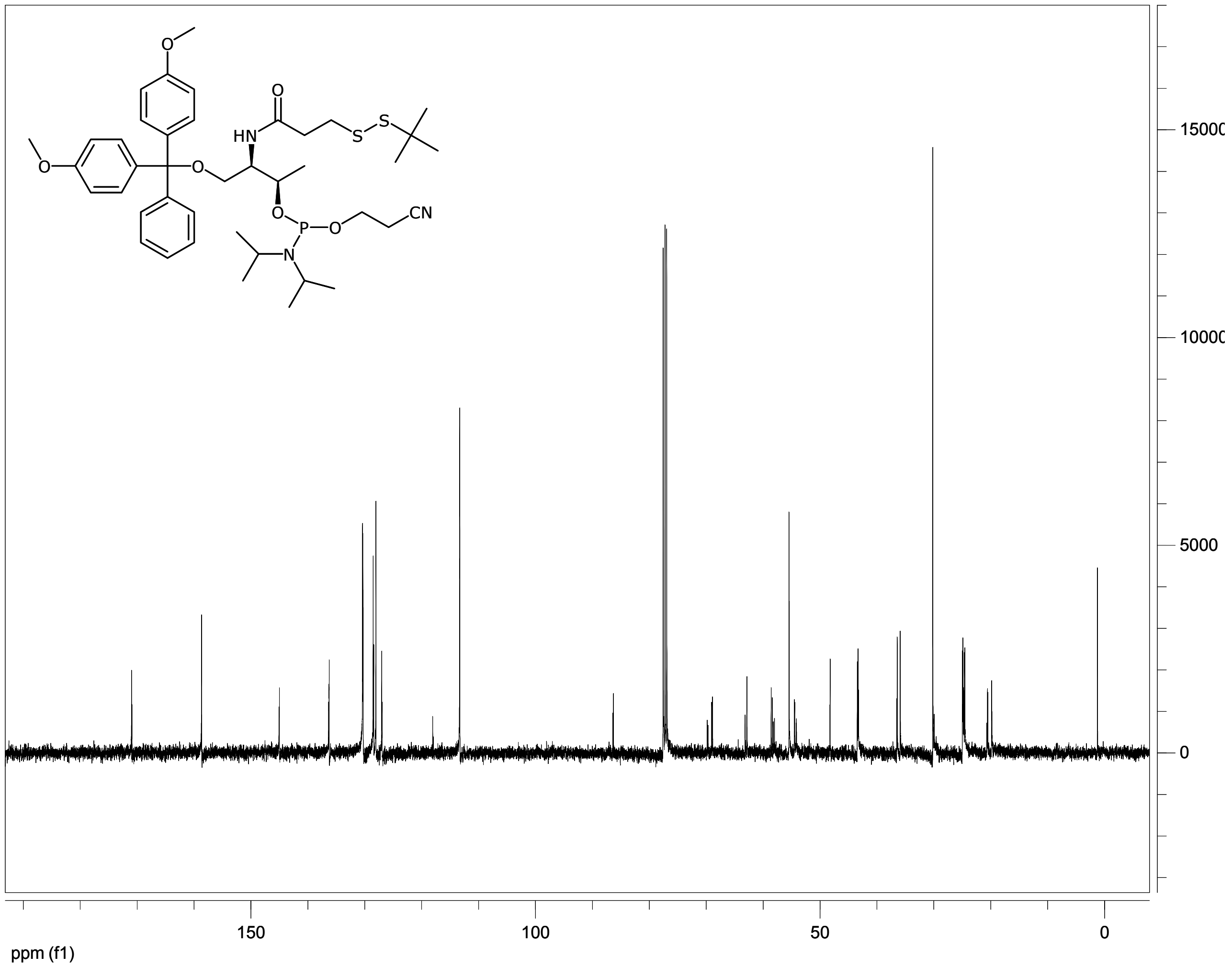

$^{31}$P-NMR spectrum of compound **6**.

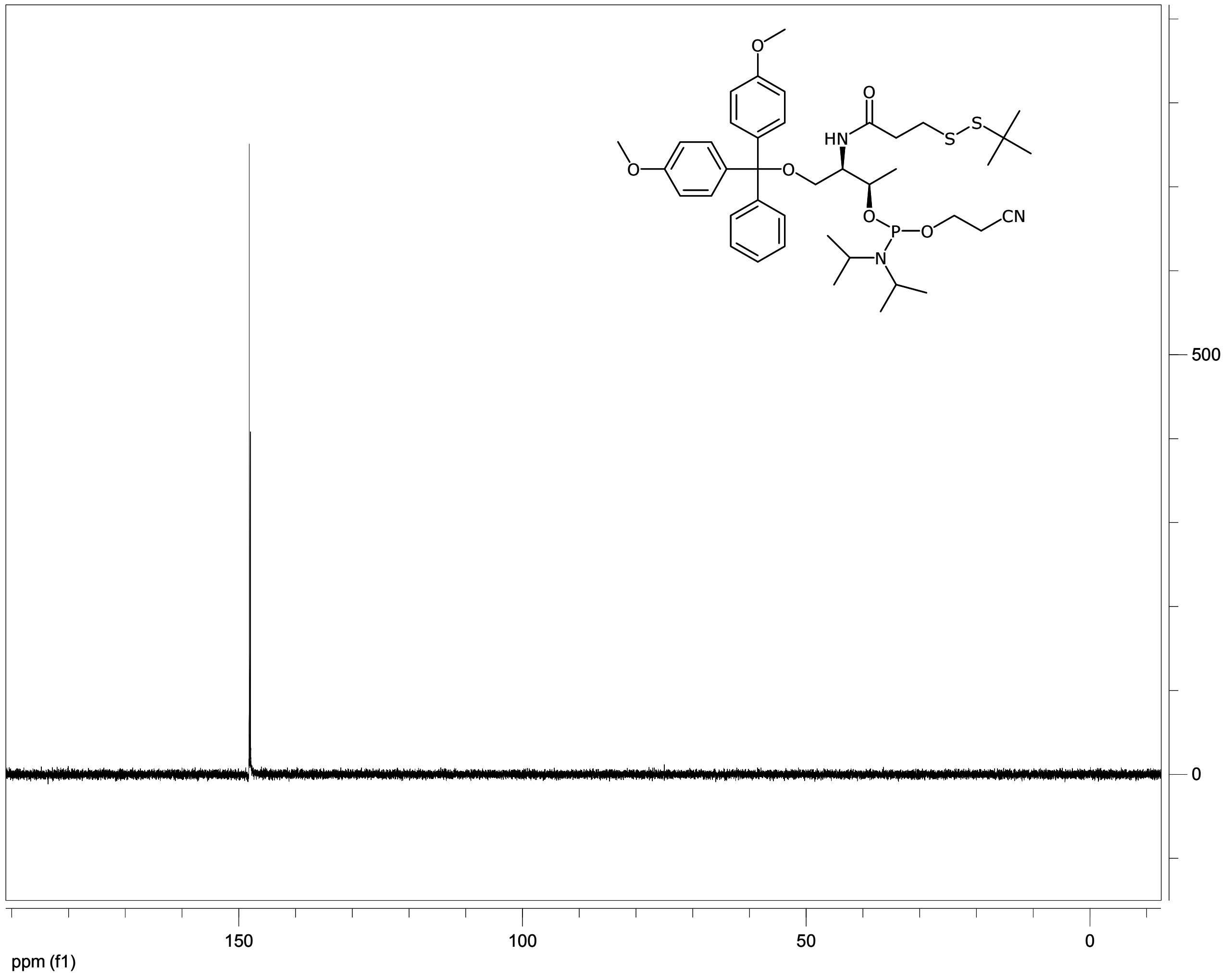

Supplement: Supplementary file 1 [file molecules-17-10026-s001.pdf]
